# Targeting miRNA‐1a and miRNA‐15b: A Novel Combinatorial Strategy to Drive Adult Cardiac Regeneration

**DOI:** 10.1002/advs.202414455

**Published:** 2025-04-03

**Authors:** Ting Yuan, Meiqian Wu, Chaonan Zhu, Hao Yu, Minh Duc Pham, Katharina Bottermann, Yijie Mao, Yue Wang, Mathias Langner, Mirko Peitzsch, Arka Provo Das, Silke Kauferstein, Jonathan Ward, Peter Mirtschink, Andreas Michael Zeiher, Stefanie Dimmeler, Jaya Krishnan

**Affiliations:** ^1^ Department of Medicine Cardiology, Goethe University Hospital 60590 Frankfurt Germany; ^2^ Institute for Cardiovascular Regeneration Centre for Molecular Medicine Goethe University Frankfurt am Main 60590 Frankfurt am Main Germany; ^3^ Cardiopulmonary Institute 60590 Frankfurt Germany; ^4^ DZHK (German Centre for Cardiovascular Research) Partner Site RheinMain 60590 Frankfurt am Main Germany; ^5^ Jilin Provincial Key Laboratory of Livestock and Poultry Feed and Feeding in the Northeastern Frigid Area College of Animal Sciences Jilin University Changchun 130062 China; ^6^ Genome Biologics Theodor‐Stern‐Kai 7 60590 Frankfurt am Main Germany; ^7^ Institute for Pharmacology Medical Faculty and University Hospital Düsseldorf Heinrich‐Heine‐University Düsseldorf 40001 Düsseldorf Germany; ^8^ Institute for Clinical Chemistry and Laboratory Medicine University Hospital Dresden Fetscherstasse 74 01307 Dresden Germany; ^9^ Department of Legal Medicine University Hospital Frankfurt Goethe University 60590 Frankfurt am Main Germany

**Keywords:** cardiac organoids, cardiomyocyte proliferation, microRNAs, myocardial infarction

## Abstract

Despite its promise, cardiac regenerative therapy remains clinically elusive due to the difficulty of spatio‐temporal control of proliferative induction, and the need to coordinately reprogram multiple regulatory pathways to overcome the strict post‐mitotic state of human adult cardiomyocytes. To address this unmet therapeutic need, a combinatorial miRNA interference screen is performed specifically targeting cardiac‐predominant miRNAs regulating key aspects of cardiomyocyte mitotic induction to cell‐cycle completion in neonatal rat cardiomyocytes. In doing so combinatorial interference of miRNA‐1a and miRNA‐15b (LNA‐1a/15b) is identified as drivers of adult cardiomyocyte proliferation. Due to miRNA‐1a/15b function on multiple processes modulating adult cardiomyocyte mitosis, its inhibition augmented adult cardiomyocyte cell‐cycle completion and daughter cell formation, and improved contractility in 3D human cardiac organoids, and in a mouse model of ST‐segment elevation myocardial infarction. Due to the cardiac‐restricted pattern of miRNA‐1a/15b expression, this strategy provides a feasible means for specific cardiomyocyte proliferative induction with minimal risk of neoplasm formation and off‐target toxicity. The approach further highlights an underutilized therapeutic strategy for simultaneous co‐regulation of multiple disease pathways through combinatorial interference of miRNAs.

## Graphical abstract

The article explores a novel therapeutic strategy for cardiac regeneration by targeting miRNA‐1a and miRNA‐15b. Combinatorial inhibition of miR‐1a and miR‐15b enhances cardiomyocyte proliferation, improves heart function, and reduces fibrosis in myocardial infarction models. The study demonstrates minimal off‐target effects and suggests a clinically feasible approach using LNA‐based inhibitors to stimulate cardiac regeneration for the treatment of heart failure.

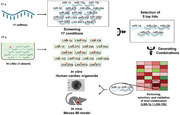

## 1 Introduction

Despite improvements in interventional and pharmacological therapies, heart failure remains a major cause of death globally. It affects 64 million people worldwide and leads to cardiovascular morbidity and mortality.^[^ ^1^ ^]^ Patients with heart failure suffer from major impairments in quality of life and poor long‐term prognosis.^[^ ^2^ ^]^ Loss of cardiomyocytes is a major hallmark of heart failure, in both congenital, age‐ and pathologic stress‐related heart failure, and is linked to an ≈25% decrease in cardiomyocytes due to cell death.^[^ ^3^ ^]^ However, unlike other tissues that can compensate cell death through proliferative induction, cardiomyocytes are post‐mitotic and have negligible capacity for proliferation, regeneration or repair to restore damaged tissue and overcome remodeling and fibrotic processes leading to heart failure and death.^[^ ^4^ ^]^ Thus, there remains a high unmet need for novel therapeutics to enhance cardiomyocyte regeneration and thereby reduce morbidity and mortality from heart failure.

In mammals, cardiomyocytes exit the cell cycle and cease proliferation in the early post‐natal period.^[^ ^5^ ^]^ Whilst evidence suggests some capacity for adult cardiomyocytes to re‐enter the cell cycle, particularly in response to myocardial insult or injury, proliferation rates are minimal with negligible physiologic or clinical benefits.^[^ ^5^, ^6^, ^7^ ^]^ Gene therapy approaches have in recent years been utilized to overcome this proliferative block with varying degrees of success.^[^ ^8^, ^9^ ^]^ Problematically, these attempts have entailed catheter‐ or intracardial injection for orthotropic delivery of therapeutics into the myocardium, which potentially limits the accessibility of such therapeutics for patients due to the complex and invasive nature of delivery.^[^ ^8^ ^]^ These invasive measures are necessary to direct therapeutic delivery to the heart and to restrict leakage of proliferation drivers to non‐cardiac tissues in order to prevent ectopic neoplasm formation. Adding to these concerns, studies indicate that proliferative induction of adult cardiomyocytes requires coordinated regulation of multiple genes to overcome the many innate barriers to adult cardiomyocyte cell‐cycle re‐entry and mitotic completion, including sarcomeric structural rigidity, stability, and density, its anti‐proliferative metabolic state and the fact that adult cardiomyocytes are prone to undergo endomitosis, rather than mitosis.^[^ ^10^ ^]^ Thus, the need to simultaneously modulate multiple pathways to induce physiologically relevant proliferative rates has raised additional technical challenges and clinical concerns.

microRNAs (miRNAs) are small non‐coding RNAs that promote mRNA degradation and translational inhibition and have been shown to play key roles in heart disease development and progression to heart failure. This is evidenced by large animal preclinical, and clinical trial data from recent and ongoing trials modulating disease‐linked miRNAs in heart disease.^[^ ^11^, ^12^, ^13^ ^]^ miRNAs function by regulating key genes involved in proliferation, hypertrophy, sarcomerogenesis, inflammation, metabolic reprogramming, and contractility.^[^ ^14^, ^15^, ^16^ ^]^ Whilst miRNAs can regulate the expression of multiple genes by binding to target transcripts to remodel the cellular transcriptome,^[^ ^17^ ^]^ it is less clear if modulation of a single miRNA alone is sufficient to re‐program all pathways necessary to effectively induce cell‐cycle re‐entry and completion of adult cardiomyocytes. Given that we were not able to identify single miRNAs capable of robustly modulating all relevant pro‐proliferative pathways in cardiomyocytes, we sought to determine if a combinatorial RNA interference (co‐RNAi) strategy would provide the necessary reprogramming impetus for adult cardiomyocyte cell‐cycle re‐entry and mitotic completion. Although largely unexplored in heart disease therapeutics, co‐RNAi strategies are widely and successfully applied in anti‐viral and cancer therapy, wherein combinatorial targeting of multiple key genes serves to better overcome phenotypic and genomic evolution to increase therapeutic robustness and efficacy.^[^ ^18^, ^19^, ^20^ ^]^ Considering precedent for successful clinical safety and efficacy of miRNA targeting therapies, we rationalized that the discovery of combinatorial targeting of cardiac‐predominant miRNAs would serve to confer a degree of cardiac‐specific therapeutic targeting and provide a feasible route for clinical translation–but also as means to coordinately reprogram the multiple pathways necessary for clinically effective adult cardiomyocyte regeneration.

To this end, we performed in silico expression and pathway connectivity analysis to identify miRNAs predominantly expressed in the heart with the capacity to modulate pro‐proliferative pathways in cardiomyocytes. These efforts led to identification of 17 miRNAs, including miRNA‐1 (miRNA‐1a in rodents), miRNA‐15a/b, miRNA‐16, miRNA‐27b, miRNA‐29a, miRNA‐34a/b/c, miRNA‐92a, miRNA‐132, miRNA‐133a, miRNA‐155, miRNA‐195a, miRNA‐208a, miRNA‐497a and miRNA‐873a as potential regulators.^[^ ^21^ ^]^ Utilizing a novel combinatorial RNAi screening methodology wherein the effects of single miRNA interference were compared on the background of interference of all other miRNAs, we identified a common core combination of miRNAs whose inhibition induced cardiomyocyte proliferation in mature human 2D and 3D in vitro cardiac ischemic models. We tested the physiologic relevance and efficacy of combinatorial inhibition of miRNA‐1a and miRNA‐15b in driving cardiac regeneration in mice subjected to myocardial infarction (MI). miRNA‐1a/15b interference with locked nucleic acid (LNA) anti‐miRs significantly augmented cardiomyocyte proliferation, as determined by EdU incorporation, phospho‐Histone H3 staining, and Aurora B localization in newly proliferating adult daughter cardiomyocytes. The capacity of miRNA‐1a/15b to augment cardiac regeneration was further reflected by the decrease in fibrosis, improved cardiac contractility, and reduced mortality in MI mice treated with miRNA‐1a/15b LNA, but not in placebo controls. Critically, despite its pro‐proliferative effects on adult cardiomyocytes, LNA‐anti‐miR‐1a/15b (LNA‐1a/15b) did not induce neoplasm formation or neoplastic metabolic reprogramming in non‐cardiac tissue. Taken together, our data may provide a novel and clinically feasible strategy to induce adult cardiac regeneration.

## 2 Results

### 2.1 Combinatorial miRNA Interference (co‐miRNAi) Screen Identifies Co‐Operative Cardiomyocyte Pro‐Proliferative Drivers

**Figure 1:**
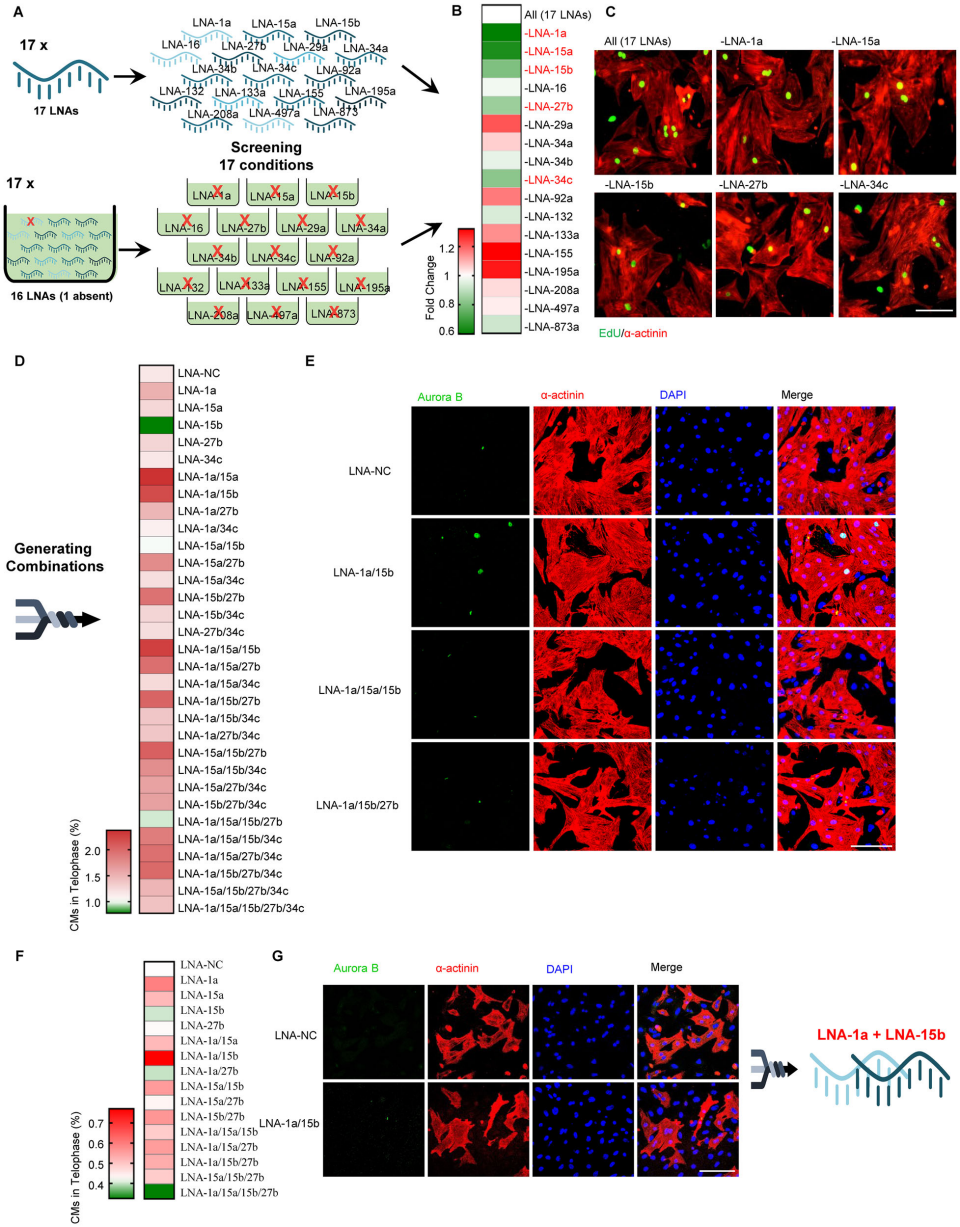
Screening of co‐miRNAi that can promote cardiomyocyte proliferation in the P1 and P7 rat cardiomyocytes. A) Schematic diagram of the research. A set of 17 LNAs have been screened and then all combinations of the top hits will be tested in the neonatal P1 and P7 rat cardiomyocytes. The effects of the best combination will then be validated in vitro and in vivo. B,C) Effect of the removal of individual LNA from the pool of 17 LNAs in the neonatal P1 rat cardiomyocytes. Neonatal P1 rat cardiomyocytes were treated with different combinations of LNAs and stained for EdU (green) and cardiomyocyte‐specific α‐actinin (red). (B) Heat map shows the fold change of EdU^+^ cells in α‐actinin^+^ cardiomyocytes. Data are normalized to All (17 LNAs). The red and green colors indicate high and low values, respectively. n = 3. (C) Representative images of EdU and α‐actinin in cardiomyocytes. Scale bar is 50 µM. D,E) Neonatal P1 rat cardiomyocytes were treated with different combinations of LNAs for 2 days, and then cardiomyocytes were stained for Aurora B (green), cardiomyocyte‐specific α‐actinin (red), and DAPI (blue). (D) Heat map shows the quantification of the percentage of Aurora B^+^ cells in telophase in α‐actinin^+^ cardiomyocytes. The red and green colors indicate high and low values, respectively. (E) Representative images of Aurora B, α‐actinin, and DAPI in cardiomyocytes. Means of n = 3 biological replicates per group. Scale bar is 100 µm. F,G) P7 rat cardiomyocytes were treated with different combinations of LNAs for 48 h and stained for Aurora B (green) and α‐actinin (red), and DAPI (blue). (F) Heat map shows the quantification of rat α‐actinin^+^ cardiomyocytes that were Aurora B^+^ in telophase as a percentage of total α‐actinin^+^ cells. Data are normalized to LNA‐NC. The red and green colors indicate high and low expression values, respectively. n = 3. G) Representative images of Aurora B, α‐actinin, and DAPI.

Utilizing the microRNA Disease Database to identify miRNAs linked to myocardial infarction (MI) and associated with cardiac anti‐proliferative pathways,^[^ ^21^ ^]^ we identified 17 miRNAs as potential regulators of adult cardiomyocyte proliferation. These miRNAs are elevated in MI and linked to proliferative pathway suppression.^[^ ^16^, ^21^, ^22^ ^]^ All 17 miRNA were expressed at detectable levels in healthy and infarcted human heart biopsies, albeit elevated in the MI patient biopsies (Figure S1A, Supporting Information). To explore the individual and potential cooperative or synergistic consequence of combined miRNA suppression, we developed an LNA combinatorial screening assay connecting specific LNAs anti‐miRs with their capacity to induce cardiomyocyte proliferation. Starting with an initial set of LNA anti‐miRs targeting all 17 miRNAs, we systematically removed one LNA (targeting a specific miRNA) from the pool of 17 LNAs, to determine how the exclusion of a single LNA (targeting one miRNA) would affect cardiomyocyte proliferation. By comparing proliferation rates in samples lacking the single LNA anti‐miR against the reference control containing all 17 LNA anti‐miRs, this assay identifies individual LNAs that function to drive cardiomyocyte proliferation. This strategy and the downstream steps of the workflow are depicted in **Figure** **1**.

We first confirmed that simultaneous interference of all 17 miRNAs by LNA anti‐miRs can lead to effective inactivation of all 17 target miRNAs in neonatal P1 rat cardiomyocytes (Figure S1B, Supporting Information). Next, we initiated the combinatorial LNA anti‐miR screen utilizing 5‐Ethynyl‐2′‐deoxyuridine (EdU)‐incorporation as an initial readout for proliferation, with co‐staining for the cardiomyocyte‐specific marker, sarcomeric α‐actinin to facilitate identification of cycling cardiomyocytes (Figure 1B,C). Strikingly, the exclusion of LNA anti‐miRs targeting miRNA‐1a, miRNA‐15a, miRNA‐15b, miRNA‐27b, and miRNA‐34c led to a reduction in cardiomyocyte proliferation of more than 15% – indicative of potential cooperative function with other LNA miRNAs in driving cardiomyocyte proliferation.

To confirm these findings, we reverted to screening LNAs targeting miRNA‐1a, miRNA‐15a, miRNA‐15b, miRNA‐27b, and miRNA‐34c both individually, and in all possible permutations in P1 rat cardiomyocytes. Whilst EdU serves as an important maker for S‐phase DNA replication, it precludes the determination of cell‐cycle completion and daughter cell formation.^[^ ^23^ ^]^ Hence, we specifically quantified Aurora B staining at telophase as a readout for completed cardiomyocyte proliferation (Figure S2A, Supporting Information). Telophase is the final phase of mitosis, during which duplicated genetic material carried in the nucleus of a parent cell is separated between two daughter cells. We assessed Aurora kinase B localization at telophase in 31 individual and combinatorial conditions corresponding to the 5 candidate LNA anti‐miRs targeting miRNA‐1a, miRNA‐15a, miRNA‐15b, miRNA‐27b, and miRNA‐34c. Cardiomyocyte proliferation was assessed by co‐localization of the contractile ring and midbody Aurora kinase B staining, with sarcomeric α‐actinin and DAPI counterstains to mark cardiomyocytes and their nuclei, respectively (Figure 1D,E; Figure S2B, Supporting Information). These analyses revealed that treatment with 3 specific LNA combinations, comprising LNA anti‐miRs targeting miRNA‐1a and miRNA‐15b (LNA‐1a/15b), miRNA‐1a, miRNA‐15a and miRNA‐15b (LNA‐1a/15a/15b), and miRNA‐1a, miR15a and miRNA‐27b (LNA‐1a/15a/27b), resulted in consistently increased numbers of cardiomyocytes in telophase compared to control scrambled LNA (LNA‐NC) treated cells (Figure 1D,E; Figures S2B and S3A–C, Supporting Information). In accord, all miRNAs targeted within the respective combinations were effectively repressed (Figure S3D–F, Supporting Information).

The early neonatal mouse heart has some proliferative and regenerative response, but this capacity is lost from P7.^[^ ^24^ ^]^ Thus, to determine the impact of the identified LNA miRNA combinations in post‐mitotic cardiomyocytes, all combinations of LNAs targeting miRNA‐1a, miRNA‐15a, miRNA‐15b, and miRNA‐27b were screened in P7 rat cardiomyocytes. Utilizing Aurora B staining at telophase as readout, the specific combination of LNAs targeting miRNA‐1a and miRNA‐15b (LNA‐1a/15b) proved most effective at inducing proliferation in post‐mitotic cardiomyocytes (Figure 1F,G; Figure S2C, Supporting Information). Consistent with previous findings, LNAs targeting miRNA‐1a and miRNA‐15b led to efficient target knockdown (Figure S4A, Supporting Information). Thus, combinatorial interference of miRNA‐1a/15b drives post‐mitotic cardiomyocyte proliferation and cell‐cycle completion in rat cardiomyocytes in vitro. To further confirm that the proliferative responses detected as a result of combinatorial inhibition of miRNA‐1a/15b were indeed originating from cardiomyocytes, and from not a hyper proliferative cell‐type such as cardiac fibroblasts, we assessed the effects of miRNA‐1a/15b LNA in cardiac fibroblasts. As shown in Figure S5A (Supporting Information), B, LNA‐anti‐miR1a/15b had negligible impact on fibroblasts proliferation. Taken together, these data suggest a degree of cell‐type specificity in LNA ‐miR1a/15b function in proliferative induction.

### 2.2 Combinatorial miRNA‐1a/15b Interference Drives Adult Cardiomyocyte Proliferation and Maintenance of Cardiac Function in Pathology

The pro‐proliferative impact of miRNA‐1a/15b function in vitro led us to explore its potential therapeutic utility in vivo. To that end, we assessed the effects of LNA‐1a/15b treatment in a mouse model of ST‐Elevation Myocardial Infarction (STEMI). STEMI is characterized by the development of acute myocardial infarction as a result of decreased coronary blood flow, accounting for ≈40% of all myocardial infarction presentations in hospitals.^[^ ^25^, ^26^ ^]^

We first assessed the efficiency of LNAs targeting miRNA‐1a and miRNA‐15 both individually and in combination in adult mice, as depicted in Figure S6A (Supporting Information). Both individual and combinatorial delivery of LNAs targeting miRNA‐1a and miRNA‐15b led to effective target suppression (Figure S6B–D, Supporting Information). Whilst depletion of miRNA‐1a or miRNA‐15b in mouse hearts led to a slight but statistically insignificant increase in proliferation, combinatorial depletion of both miRNA‐1a and miRNA‐15b significantly induced cardiomyocyte proliferation as shown by increased pHH3 (Figure S6E,F, Supporting Information).

**Figure 2:**
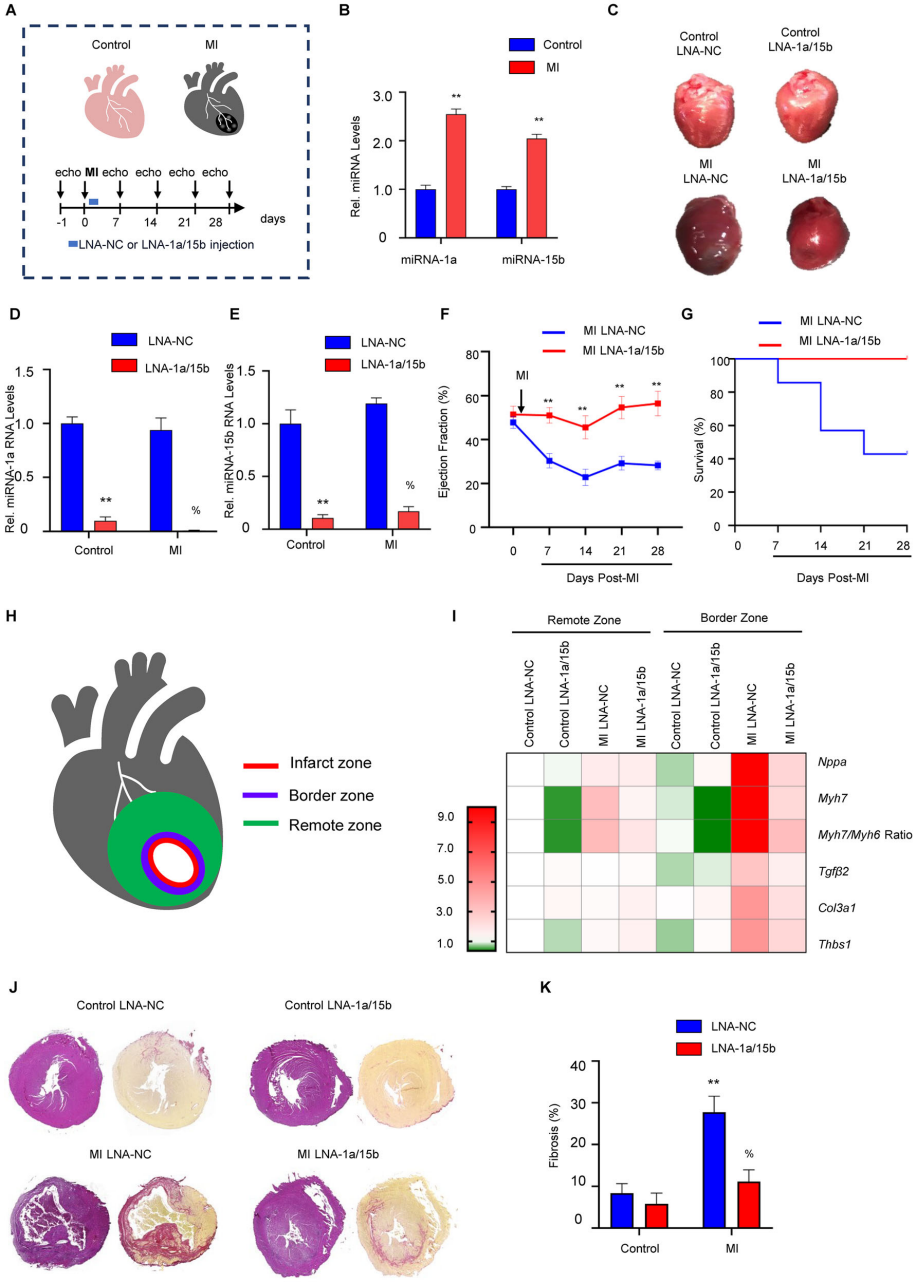
The effects of combinatorial miRNA‐1a/15b interference on myocardial infarction‐induced cardiac damage in vivo. A) Schematic representation of the experimental timeline of C57BL/6J mice subjected to MI surgery. The LNA‐1a/15b or LNA‐NC was injected at 4 h after MI surgery, and echocardiography measurement was performed on days 0, 7, 14, 21, and 28. The mouse hearts were harvested on day 28 after MI surgery. A subset of heart biopsies were harvested at 8 hours post‐MI, serving as an internal control for the pathologic stress‐induced upregulation of miR‐1a and miR‐15b. B) Relative miRNA expression in heart border zone biopsies from mice from mice 8 hours post‐MI and healthy controls. Data are expressed as means ± SEM; n = 3 mice per group; ^**^ *P* < 0.01 versus control. Two‐tailed unpaired t‐test. C) Whole hearts harvested from mice. D) Relative expression of miRNA‐1a in remote zone of mice subjected to control or MI surgery. Data are expressed as means ± SEM; n = 4 mice per group. ^**^ *P* < 0.01 versus Control LNA‐NC, %*P* < 0.05 versus MI LNA‐NC. Two‐tailed unpaired t‐test. E) Relative expression of miRNA‐15b in remote zone of mice subjected to control or MI surgery. Data are expressed as means ± SEM; n = 4 mice per group; ^**^ *P* < 0.01 versus Control LNA‐NC, %*P* < 0.05 versus MI LNA‐NC. Two‐tailed unpaired t‐test. F) Longitudinal monitoring of cardiac % Ejection fraction (%EF). Data are expressed as means ± SEM; N = 4/5 mice per group; ^**^ *P* < 0.01 versus MI LNA‐NC. Two‐tailed unpaired *t*‐test. G) Survival curve in MI group. N = 4/5 mice per group. H) The mouse heart was divided into the Remote and Border zone, as depicted. I) Heat map shows the mRNA expression levels of pathological remodeling marker genes (*Nppa*, *Myh7*, and *Myh7/Myh6 ratio*) and fibrotic remodeling marker genes (*Tgfβ2*, *Col3a1*, and *Thbs1*) after LNA‐NC or LNA‐1a/15b injection and MI injury in the Remote and Border zone. Data was normalized to Control LNA‐NC. The red and green colors indicate high and low expression values, respectively. n = 4 mice per group. Two‐tailed unpaired *t*‐test. J) Representative hematoxylin and eosin (HE) staining and picrosirius red stained heart sections at 28‐days post MI surgery. K) Quantitative cardiac fibrosis analysis. Data are expressed as means ± SEM; n = 4 mice per group; ^**^ *P* < 0.01 versus Control LNA‐NC, %*P* < 0.05 versus MI LNA‐NC. Two‐tailed unpaired *t*‐test. MI, Myocardial Infarction.

We next examined if inhibition of miRNA‐1a/15b could similarly increase cardiomyocyte proliferation in response to a mouse STEMI model (**Figure** **2**A). MI was induced by permanent ligation of left anterior descending coronary artery, resulting in elevated expression of miRNA‐1a and miRNA‐15b 8 h post‐MI in the border zone (Figure 2B). Mice were treated with LNA‐1a/15b four h post‐surgery to mimic the average duration that post‐STEMI patients receive treatment.^[^ ^25^, ^26^ ^]^ At 28 days post‐MI surgery the experiment was terminated due to the decline in cardiac function and increased mortality in mice subjected to MI and treated with control non‐silencing LNAs (LNA‐NC) (Figure 2C). LNA‐1a/15b treatment effectively suppressed RNA levels of miRNA‐1a and miRNA‐15b in remote zone biopsies of the respective mice (Figure 2D,E). Echocardiographic measure of ejection fraction (EF), as a readout for cardiac contractile function, was rapidly reduced in LNA‐NC treated mice subjected to MI concomitant to increased mortality (Figure 2F,G). In contrast, mice treated with LNA‐1a/15b largely maintained cardiac function and organismal viability despite MI intervention (Figure 2F,G). To ascertain the effects of LNA‐1a/15b treatment in mice subjected to MI, the heart was first divided into remote and border zones (Figure 2H). As the detected rescue and maintenance of healthy cardiac in LNA‐1a/15b treated mice would predict reduced expression of pathologic hypertrophy markers, we examined the expression of *atrial natriuretic peptide* (*Nppa*), *beta‐myosin heavy chain 6* (*Myh6*) and *beta‐myosin heavy chain 7* (*Myh7*) (Figure 2I). *Nppa* and *Myh7* RNA levels were reduced after LNA‐1a/15b injection in the border zone compared to the control LNA‐NC injection (Figure 2I). Similarly, MI‐induced *Myh7*:*Myh6* ratio was reduced by combinatorial inhibition of miRNA‐1a and miRNA‐15b, indicative of suppression of the pathologic hypertrophic gene program induction in LNA‐1a/15b treated hearts (Figure 2I). The remote zone serves as an internal tissue control due to its distance from the infarct site and is thus less affected by tissue damage caused by MI (Figure 2H). Consistent with this, LNA‐1a/15b treatment primarily impacted hypertrophic marker expression in border zone biopsies, but not in the largely unaffected remote zone (Figure 2I).

Maintenance of cardiac function and the absence of mortality in MI mice treated with LNA‐1a/15b indicates a possible attenuation of cardiac fibrosis and stiffness in these mice. Thus, we profiled key fibrotic marker genes including *transforming growth factor β2* (*Tgfβ2*), *Collagen type III alpha 1 chain* (*Col3a1*), and *thrombospondin 1* (*Thbs1*). As noted in Figure 2I, fibrotic marker gene expression was significantly reduced in MI mice treated with LNA‐1a/15b compared to LNA‐NC controls. Consistent with the negligible impact of the infarct on the remote zone, fibrotic marker gene expression in the remote zone was indistinguishable between control and MI mice, regardless of the treatment regime (Figure 2I). Finally, we stained sections of the respective hearts with Picrosirius Red, an established marker for tissue fibrosis.^[^ ^27^ ^]^ As noted in Figure 2J,K, LNA‐1a/15b treated MI mice exhibited considerably less cardiac fibrosis compared to LNA‐NC treated controls. Consistent with this observation, staining for the apoptosis marker cleaved‐Caspase 3, revealed increased cell death in mouse LNA‐NC‐treated hearts subjected to MI, but not in hearts treated with LNA‐1a/15b (Figure S7A, Supporting Information). In sum, LNA‐1a/15b treatment significantly attenuated the development of cardiac fibrosis, and its progression to heart failure and death in a mouse model of STEMI.

### 2.3 Combinatorial miRNA‐1a/15b Interference Specifically Drives Cardiomyocyte Proliferation In Vivo with Minimal Off‐Target Effects

**Figure 3:**
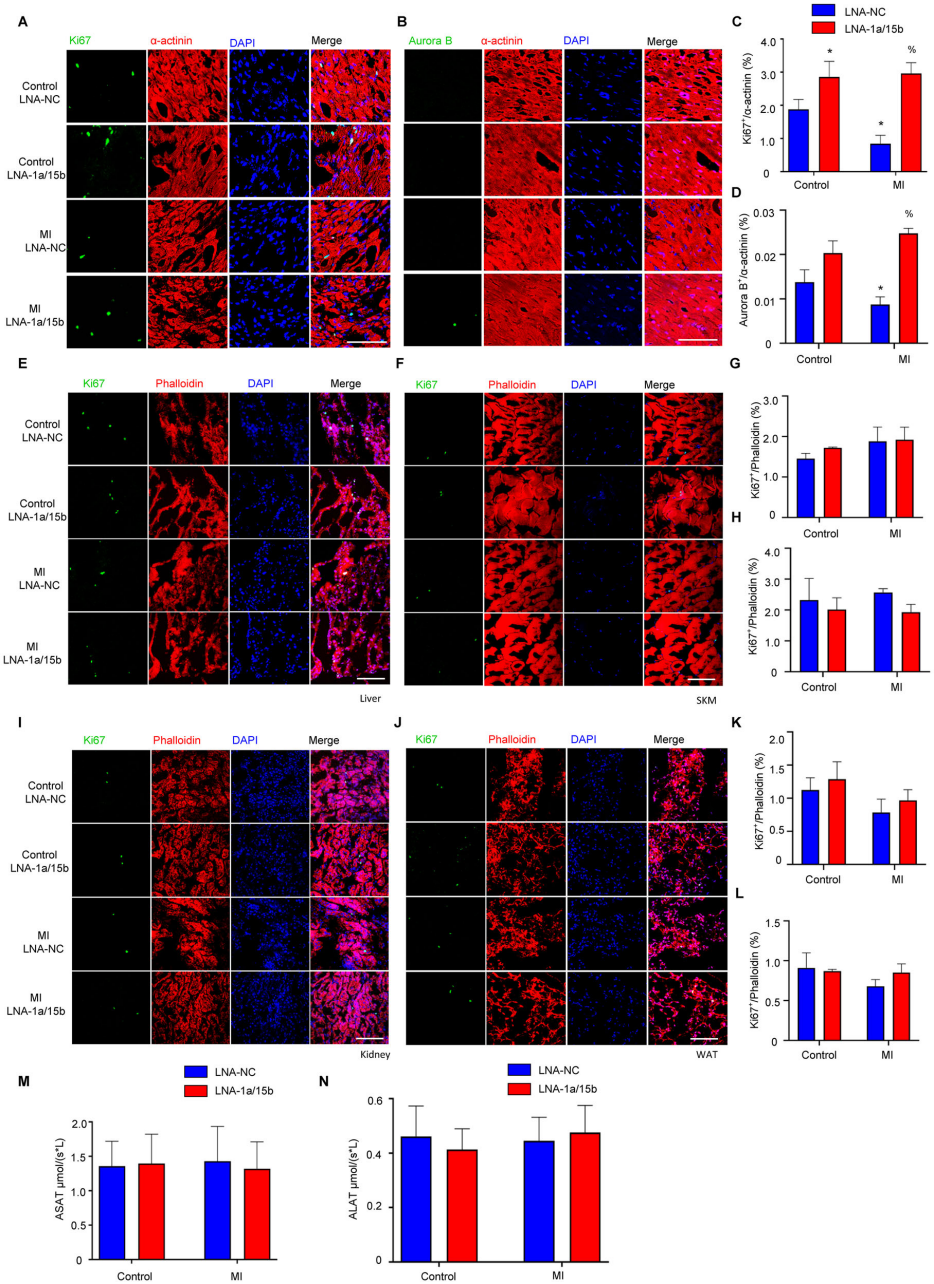
The effects of combinatorial miRNA‐1a/15b interference on proliferation in cardiac tissue and in non‐cardiac tissues in vivo. A) Representative immunofluorescence images of Ki67 on heart sections of adult hearts injected with LNA‐1a/15b or control 28‐days post MI injury. Ki67 labels proliferating cells (green); cardiomyocyte‐specific α‐actinin (red) and DAPI (Blue). B) Representative immunofluorescence images of Aurora B on heart sections of adult hearts injected with LNA‐1a/15b or control 28‐days post MI injury. Aurora B labels proliferating cells (green); cardiomyocyte‐specific α‐actinin (red) and DAPI (Blue). C) Quantification of percentages of Ki67^+^ cardiomyocytes. Data are expressed as means ± SEM; N = 3 mice per Control group, n = 5 mice per MI group; ^*^ *P* < 0.05 versus Control LNA‐NC, %*P* < 0.05 versus MI LNA‐NC. Two‐tailed unpaired t‐test. Scale bar is 100 µm. D) Quantification of percentages of Aurora B^+^ cardiomyocytes. Data are expressed as means ± SEM; N = 3 mice per Control group, N = 5 mice per MI group; %*P* < 0.05 versus MI LNA‐NC. Two‐tailed unpaired t‐test. Scale bar is 100 µm. E) Representative images of Ki67 (green), Phalloidin (red), and DAPI (blue) in liver. Scale bar is 100 µm. F) Representative images of Ki67 (green), Phalloidin (red), and DAPI (blue) in SKM. Scale bar is 100 µm. G) Quantification of percentages of Ki67^+^ liver cells. Data are expressed as means ± SEM; n = 3 mice per group. Two‐tailed unpaired t‐test. H) Quantification of percentages of Ki67^+^ SKM cells. Data are expressed as means ± SEM; n = 3 mice per group. Two‐tailed unpaired t‐test. I) Representative images of Ki67 (green), Phalloidin (red), and DAPI (blue) in kidney. Scale bar is 100 µm. J) Representative images of Ki67 (green), Phalloidin (red), and DAPI (blue) in WAT. Scale bar is 100 µm. K) Quantification of percentages of Ki67^+^ kidney cells. Data are expressed as means ± SEM; n = 3 mice per group. Two‐tailed unpaired t‐test. L) Quantification of percentages of Ki67^+^ WAT cells. Data are expressed as means ± SEM; n = 3 mice per group. Two‐tailed unpaired t‐test. SKM, Skeletal Muscle; WAT, White Adipose Tissue. M) ASAT and N) ALAT levels in plasma of C57BL/6J mice injected with wither LNA‐1a/15b or Control LNA‐NC 28‐days post MI injury. Data are expressed as means ± SEM; n = 5‐6 mice per group. MI, Myocardial Infarction; ASAT, Aspartate aminotransferase; ALAT, Alanine aminotransferase.

To ascertain if the beneficial effects of combinatorial miRNA‐1a and miRNA‐15b depletion are linked to adult cardiomyocyte proliferation, we examined the distribution of the mitotic S‐phase marker Ki67 and the metaphase marker phosphorylated Histone H3 (pHH3) in heart biopsies of the respective groups. Increased Ki67 and pHH3 signal was detected in cardiomyocytes of both sham and MI LNA‐1a/15b treated mice (**Figure** **3**A–D; Figure S8A,B, Supporting Information), with greater levels of Ki67 and pHH3 induction observed in MI operated mice. Strikingly, when the respective biopsies were assessed for expression of the cytokinesis marker Aurora B, as a readout for cell‐cycle completion and daughter cell formation, we only detected a significant increase in the fraction of Aurora B^+^ cardiomyocytes in hearts of mice subjected to MI and treated with LNA‐1a/15b (Figure 3B,D).

LNAs are delivered systemically and can detrimentally affect non‐target tissues. This is particularly relevant in regenerative‐ or proliferative‐ type therapies where proliferation activators targeting specific cell‐types, may in parallel induce neoplasm formation due to mitotic activation in non‐target cells. Thus, a degree of cell‐type specificality in therapeutic response would be prudent. In Figure S5A,B (Supporting Information), we demonstrated a lack of LNA‐1a/15b impact on cardiac fibroblasts proliferation. To extend these findings, we asked how LNA‐1a/15b inactivation would impact tissue proliferation of key non‐cardiac cell‐types in mice. As noted in Figures 3E–L and S9A–H (Supporting Information), negligible changes in cell proliferation, as determined by Ki67 and pHH3 staining, was detected in tissues including liver, skeletal muscle (SKM), kidney and white adipose tissue (WAT) of mice subjected to sham or MI surgery, in the absence or presence of LNA‐1a/15b treatment.

Changes in cell and tissue metabolism is another important feature of neoplastic transformation and are broadly characterized by a shift from glucose and fatty acid oxidation as the primary mode of ATP generation to glycolysis, a process less efficient in ATP generation but advantageous for the generation of nucleotides, amino acids and phospholipids necessary building‐blocks of cell proliferation.^[^ ^28^, ^29^ ^]^ Given this, we performed metabolomic analysis on non‐cardiac tissue namely the liver, lung, kidney, spleen and white adipose tissue (WAT) (Figure S10A–E, Supporting Information). Principal Component Analysis (PCA) revealed broad and overlapping signals distribution across the respective groups in liver, lung, kidney, and spleen biopsies, suggestive of insignificant deviation from Control LNA‐NC treated mice. In contrast, analysis of WAT biopsies from the respective groups revealed unique signal clustering of biopsies derived from LNA‐NC mice subjected to MI compared to the other groups. Importantly, WAT biopsies harvested from mice subjected to MI and treated with LNA‐1a/15b exhibited pronounced signal overlap with Control LNA‐NC treated tissue (Figure S10A–E, Supporting Information). Taken together, combinatorial inhibition of miRNA‐1a and miRNA‐15b caused negligible off‐target effects on non‐cardiac tissue metabolism and proliferation rates.

Finally, to understand any possible effect of LNA‐1a/15b therapy on systemic toxicity, we assessed circulating levels of biomarker enzymes Aspartate aminotransferase (ASAT) and Alanine aminotransferase (ALAT).^[^ ^30^ ^]^ While ASAT is commonly associated with liver and heart damage, it can also be found in other tissues, including skeletal muscle and kidneys. ALAT is primarily found in hepatocytes. Upon tissue damage due to drug toxicity, these enzymes are released into the blood. Upon tissue damage due to drug toxicity, release these enzymes into the blood. Thus, circulating ASAT and ALAT levels in the blood directly correlates with tissue damage and toxicity.^[^ ^31^ ^]^ As noted in Figure 3M,N, LNA‐1a/15b had minimal impact on circulating ASAT and ALAT levels compared to controls, indicative of an acceptable safety profile. In sum, these data indicate that LNA‐1a/15b treatment is non‐toxic and provokes minimal tissue off‐target effects.

### 2.4 Combinatorial miRNA‐1/15b Interference Enhances Cardiomyocyte Proliferation and Function in Human Cardiac Organoids

**Figure 4:**
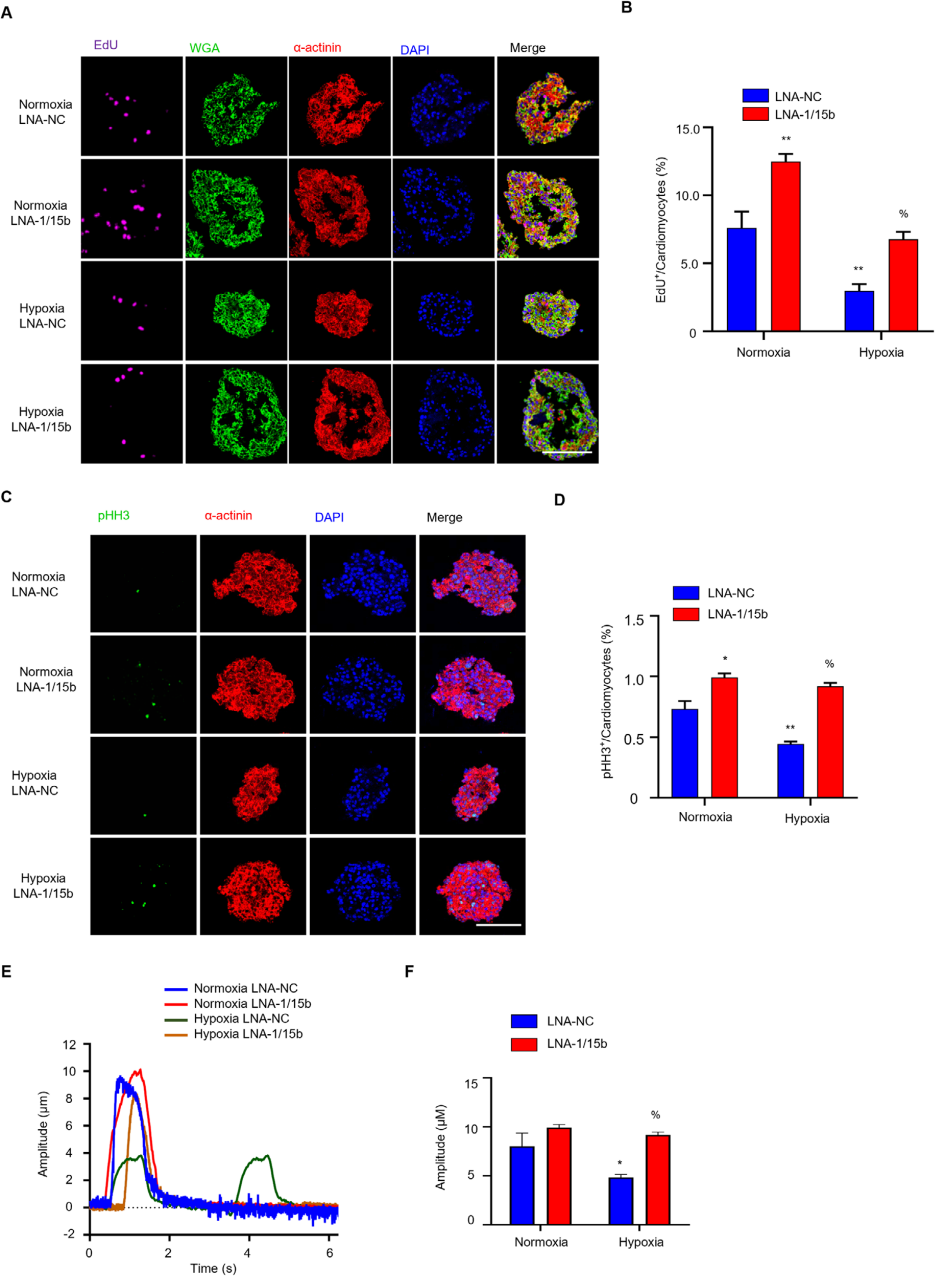
The effects of combinatorial miRNA‐1a/15b interference on cardiomyocyte proliferation and cardiac function in human cardiac tissue mimetics. A) Representative immunofluorescence images of EdU incorporation on sections of 40‐day‐old human cardiac mimetics treated with LNA‐1a/15b or LNA‐NC in both normoxia and hypoxia. EdU labels proliferating cells (Magenta); Wheat‐Germ‐Agglutinin (WGA) marks cell surfaces (green); cardiomyocyte‐specific α‐actinin (red) and DAPI (Blue). B) Quantification of percentages of EdU^+^ cardiomyocytes. Data are expressed as means ± SEM; n = 3 per group; ^**^ *P* < 0.01 versus Normoxia LNA‐NC, %*P* < 0.05 versus Hypoxia LNA‐NC. Two‐tailed unpaired t‐test. Scale bar is 100 µm. C) Representative immunofluorescence images of pHH3 on sections of 40‐day‐old human cardiac mimetics treated with LNA‐1a/15b or LNA‐NC in both normoxia and hypoxia. pHH3 labels proliferating cells (Magenta); cardiomyocyte‐specific α‐actinin (red) and DAPI (Blue). Scale bar is 100 µm. D) Quantification of percentages of pHH3^+^ cardiomyocytes. Data are expressed as means ± SEM; n = 3 per group; ^**^ *P* < 0.01 versus Normoxia LNA‐NC, %*P* < 0.05 versus Hypoxia LNA‐NC. Two‐tailed unpaired t‐test. E) Representative traces of contractile human cardiac mimetics. Human cardiac mimetics were treated with LNA‐1a/15b or LNA‐NC for 3 days in both normoxia and hypoxia, and then the contractility assays were performed by determining the amplitude peak of contracting human cardiac mimetics. F) Quantification of the amplitude peak of contracting human cardiac mimetics. Data are expressed as means ± SEM; 4 – 5 organoids per group; *P < 0.05 versus Normoxia LNA‐NC, %*P* < 0.05 versus Hypoxia LNA‐NC. Two‐tailed unpaired t‐test.

In order to understand the potential of LNA‐1a/15b therapy for clinical translation, we first assessed the effects of LNA‐1/15b in the human induced pluripotent stem cells‐derived cardiomyocytes (hiPSC‐CMs) cultured under control normoxia or in hypoxia (1%O_2_) as an in vitro model for myocardial infarction.^[^ ^32^ ^]^ LNA‐1/15b treatment effectively suppressed RNA levels of miRNA‐1 and miRNA‐15b in the hiPSC‐CMs (Figure S11A,B, Supporting Information) in normoxia and hypoxia. As observed in Figure S11C,D (Supporting Information), LNA‐1/15b treatment led to increased Aurora B signal, indicative of a conserved function for LNA‐1/15b in driving mature human cardiomyocyte proliferation and cell‐cycle completion. Next, we utilized human iPSC‐derived 3D cardiac tissue mimetics to determine the proliferative impact of LNA‐1/15b treatment in tissue. Utilizing a variation of a previously established 3D cardiac mimetic model with the inclusion of a maturation phase,^[^ ^33^, ^34^, ^35^ ^]^ we were able to generate adult cardiac mimetics exhibiting spontaneous beating characteristics and gene expression profiles characteristic of adult tissue^[^ ^36^ ^]^ (Figure S12A–H and Video S1, Supporting Information). In this setting, cardiac mimetics were cultured under control normoxia or in hypoxia, treated with LNA‐NC or LNA‐1/15b, and assessed for proliferation by EdU‐incorporation (as readout for S‐Phase entry and DNA replication) and pHH3 staining (**Figure** **4**A–D). As observed in the mouse STEMI model, a similar pattern of proliferative induction by LNA‐1/15b was detected in both control normoxia and hypoxic conditions, with elevated levels of proliferation observed in hypoxia. Consistent with previous data, LNA‐1/15b treatment effectively suppressed RNA levels of miRNA‐1 and miRNA‐15b in human cardiac mimetics (Figure S13A,B, Supporting Information). Furthermore, as observed in the mouse studies, LNA‐1/15b treatment of human organoids subjected to severe hypoxic stress prevented the onset of contractile dysfunction (Figure 4sE,FZ; Figure S13C,D, Supporting Information). Contractility was determined by determining the peak amplitude of contracting cardiac organoids (Figure 4E) and calcium flux measurements (Figure S13C, Supporting Information) from the respective groups, and quantified as shown in Figures 4F and S13D (Supporting Information). Cardiac organoids cultured in hypoxia revealed the expected decrease in contractile amplitude concomitant to increased cycling frequency (Figure 4E,F; Figure S13C,D, Supporting Information). However, LNA‐1/15b treatment in human cardiac mimetics rescued the loss in contractility induced by severe hypoxic stimulation (Figure 4; Figure S13C,D, Supporting Information). In sum, our data indicates that LNA‐1/15b drives mitotic entry and cell‐cycle completion in to maintain cardiac function in mature human tissue in the face of hypoxic insult.

### 2.5 Mechanistic Basis of LNA‐1a/15b Function in Cardiomyocyte Proliferative Induction

**Figure 5:**
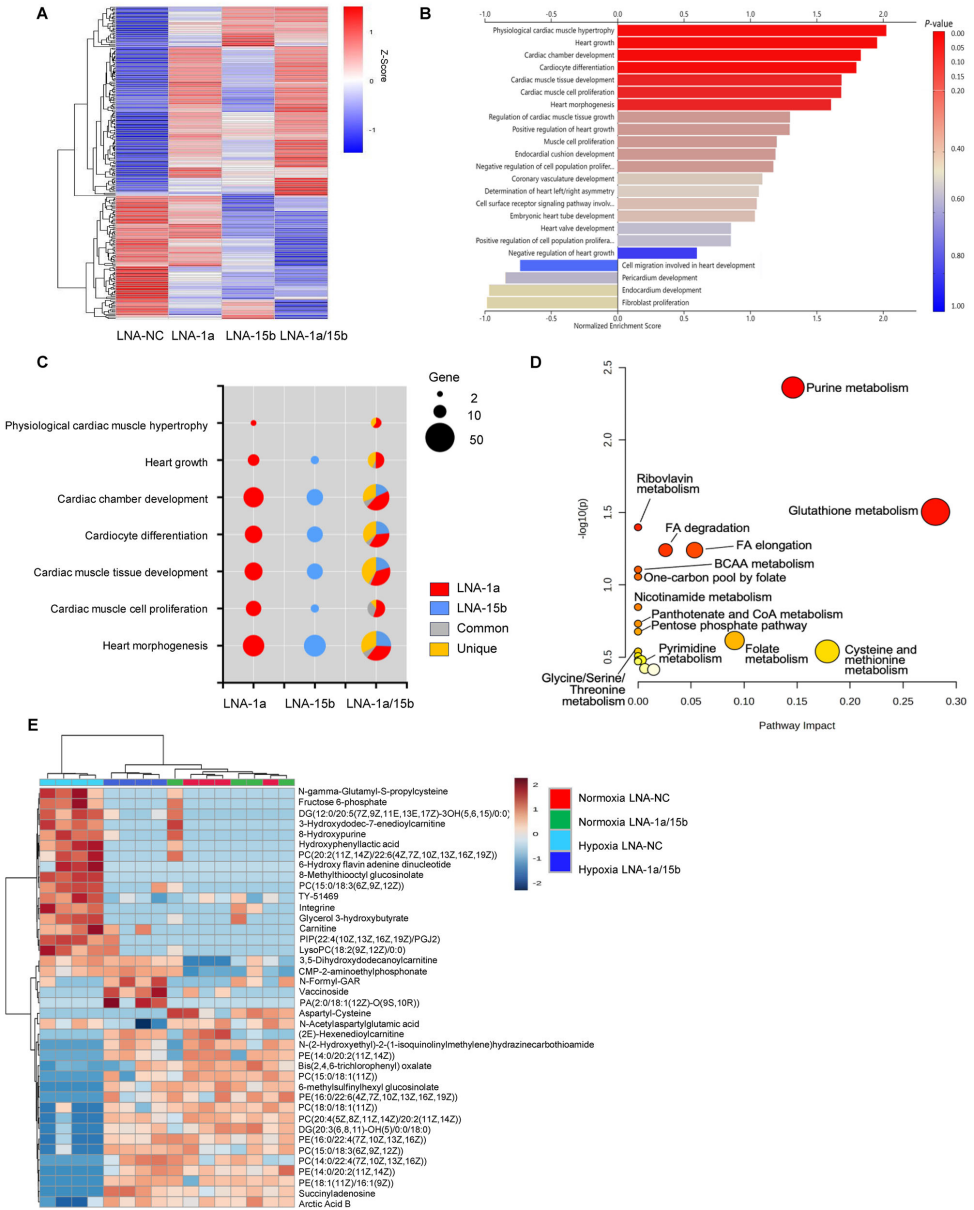
Changes in gene expression and metabolites with inhibition of miRNA‐1a and miRNA‐15b. A) The heatmap shows the variant genes. X‐axis: samples of LNA‐NC, LNA‐1a, LNA‐15b, LNA‐1a/15b; The red and blue colors indicate upregulation and downregulation, respectively. Means of n = 3 biological replicates per group. B) Gene Ontology (GO) biological process terms enriched in LNA‐1a/15b‐regulated genes. C) Top 7 GO biological process terms related to the heart development enriched in LNA‐1a, LNA‐15b, and LNA‐1a/15b. Red, genes regulated specifically by miRNA‐1a inhibition; Blue, genes regulated specifically by miRNA‐15b inhibition; grey, genes regulated in‐common by both miRNA‐1a and miRNA‐15b depletion; yellow, genes regulated unique to combinatorial inhibition of miRNA‐1a and miRNA‐15b. D) Pathway analysis bubble plot in KEGG shows the enriched metabolic pathways in P1 rat cardiomyocytes treated with LNA‐1a/15b under hypoxia. The horizontal axis is the pathway impact, which represents the importance of differential metabolites in metabolic pathways. The vertical axis represents the results of pathway enrichment analysis. The dot size represents the centrality of the metabolites involved in the corresponding metabolic pathway. The color of the dot represents the impact factor; large sizes and dark colors represent the central metabolic pathway enrichment and pathway impact values, respectively. E) Heat map of relative metabolite abundance in Neonatal P1 rat cardiomyocytes treated with either control LNA‐NC or LNA‐1a/15b, and then incubated in the hypoxia and hypoxia for 2 days. Depicted are metabolites with log2(fold change) > 0.5 compared to Normoxia LNA‐NC treatment and adjusted *p* value < 0.01 in at least one treatment group compared to the corresponding control LNA‐NC. n = 4 biological replicates per group.

Whilst the vast majority of therapeutic interventions involving miRNA inhibition rely on the targeted inactivation of a single miRNA, our screening efforts reveal that combinatorial approaches can potentially provide greater efficacy and robustness in driving adult cardiomyocyte proliferation. Further, this combinatorial effect is not restricted to rodents, rather it is conserved across species as noted by the effects of miRNA‐1a/15b inactivation in mouse, rat, and human cardiomyocytes and cardiac tissue (Figure 1D–G; Figure S6E,F, Supporting Information; Figure 3A–D; Figure S8A,B, Supporting Information; Figure 4A–D). Strikingly, due to the enrichment of miRNA‐1a and miRNA‐15b in cardiomyocytes, we detected a negligible effect of LNA‐1a/15b treatment on proliferative induction of fibroblasts, and on the heterogenous population of cells contained within non‐cardiac tissue (Figure S5A,B, Supporting Information; Figure 3E–L; Figure S9A–H, Supporting Information). The specificity of LNA‐1a/15b function is also reflected at the level of the respective non‐cardiac tissue metabolomes (Figure S10A–E, Supporting Information). Given this, we sought to understand the downstream consequence of miRNA‐1a/15b inhibition on coding gene regulators, as mediators of proliferative induction, specifically in cardiomyocytes. Thus, NRCs were treated with LNA‐NC, LNA‐1a, LNA‐15b or LNA‐1a/15b and hypoxia stimulated (to mimic the MI setting). As shown in the heatmap in **Figure** **5**A, differential gene expression analysis revealed specific changes in gene expression unique to cardiomyocytes stimulated with the combination of LNAs targeting both miR1a/15b, but not in control LNA‐NC, or in cardiomyocytes treated with LNAs targeting only miRNA‐1a or miRNA‐15b individually. Next, Kyoto Encyclopedia of Genes and Genomes (KEGG) clustering analysis revealed enrichment of genes linked to cardiac development, cell/tissue homeostasis, and cardiac growth and morphogenesis (Figure 5B; Figure S14A–G, Supporting Information). Consistent with this data, Gene Set Enrichment Analysis (GSEA) enrichment analysis of all differentially expressed genes between LNA‐NC, LNA‐1a, LNA‐15b, and LNA‐1a/15b treated groups revealed distribution into cardiac linked pathways (Figure 5C). Given that the LNA‐1a/15b combination proved most relevant by virtue of its ability to maintain cardiac function and organismal survival in the face of MI, we sub‐classified genes within the respective Gene Ontology (GO) pathways into 4 groups comprising of genes regulated specifically by miRNA‐1a or miRNA‐15b depletion, respectively (miRNA‐1a and miRNA‐15b only), genes regulated in‐common by both miRNA‐1a and miRNA‐15b depletion (Common), and differentially expressed genes specific to combinatorial inhibition of miRNA‐1a and miRNA‐15b (Unique) (Figure 5C). As expected, regulation of the vast majority of genes can be linked directly to miRNA‐1a and/or miRNA‐15b function (Figure 5C; Figure S15A–H, Supporting Information). Surprisingly, a fairly large number of differentially expressed genes could neither be linked to direct miRNA‐1a or miRNA‐15b function, based on known and predicted targets of the respective miRNAs and our expression data (Figure S15D,H, Supporting Information).^[^ ^21^ ^]^ Genes depicted in the Figure S15D (Supporting Information) heatmap encompass miRNA‐1a/15b regulated genes that are unaffected or only mildly altered (and below the significance threshold) upon treatment with miRNA‐1a LNAs or miRNA‐15b LNAs individually. Many of the genes within this Unique subgroup are characterized by a role in inducing cardiomyocyte de‐differentiation, early cardiac development and remodeling comprising of genes such as T‐Box Transcription Factor 20 (TBX20), PR‐Domain Zinc Finger Protein 16 (PRDM16), ADP‐Ribosylhydrolase Like 1 (ADPRHL1),^[^ ^37^ ^]^ Glycogen Synthase Kinase 3 Beta (GSK3B), Fibroblast Growth Factor Receptor Substrate 2 (FRS2), Sorbin And SH3 Domain Containing 2 (SORBS2), WW Domain Containing Transcription Regulator 1 (WWTR1) and SRY‐Box Transcription Factor 9 (SOX9) (Figure S15D, Supporting Information). Connected to these are modulators of sarcomerogenesis, cardiac calcium handling and contractility including Cysteine And Glycine Rich Protein 3 (CSRP3), Synaptopodin 2 Like (SYNPO2L), Myosin XVIIIB (MYO18B), Xin Actin Binding Repeat Containing 1 (XIRP1), NADPH Oxidase 4 (NOX4), Calcium Voltage‐Gated Channel Subunit Alpha1 C (CACNA1C), Mitochondrial Dynamin Like GTPase (OPA1), Ankyrin 2 (ANK2), PDZ And LIM Domain 7 (PDRM17) and Ryanodine Receptor 2 (RYR2) (Figure S15D, Supporting Information). Further, we detected upregulation of drivers of angiogenesis, lymphangiogenesis, neurogenesis, and extracellular matrix and stromal cell activators consisting of Prospero Homeobox 1 (PROX1), Neuropilin 2 (NRP2) and Transforming Growth Factor Beta 3 (TGFB3) (Figure S15D, Supporting Information). Finally, this Unique group contained general transcriptional and translational modulators, and epigenetic regulators including key factors such as AT‐Rich Interaction Domain 1A (ARID1A), Polybromo 1 (PBRM1), Mediator Complex Subunit 1 (MED1), Lysine Demethylase 6B (KDM6B), and lesser understood components including Zinc Finger MIZ‐Type Containing 1 (ZMIZ1) and Dishevelled Segment Polarity Protein 3 (DVL3) (Figure S15D, Supporting Information). Taken together, these data suggest a possible molecular synergism wherein simultaneous depletion of miRNA‐1a and miRNA‐15b facilitates the specific activation of genes, which in this context serves to provide a pro‐proliferative environment in post‐mitotic adult cardiomyocytes by co‐opting key accessory pathways driving adult cardiomyocyte de‐differentiation and plasticity, remodeling of the cardiomyocyte sarcomere and contractile machinery, concomitant to the induction of cardiac angiogenesis, neurogenesis and stromal activation to support survival of new cardiomyocytes, to enable effective adult cardiac regeneration.

Given the crucial role of the cell metabolome in enabling and facilitating cell proliferation,^[^ ^38^ ^]^ we analyzed changes in cellular metabolite composition as a result of LNA‐1a/15b treatment under basal and hypoxic stress conditions. As noted in Figure 5D,E, depletion of miRNA‐1a/15b in hypoxic cardiomyocytes led to an elevation of metabolites necessary for proliferative induction and cell‐cycle completion, including metabolites of purine and pyrimidine biosynthesis,^[^ ^39^, ^40^ ^]^ branched‐chain amino acid (BCAA) metabolites,^[^ ^28^, ^41^ ^]^ nicotinamide and riboflavin components,^[^ ^28^, ^41^ ^]^ panthotenate and co‐enzyme A metabolites necessary for acylation of metabolic intermediates,^[^ ^39^, ^42^ ^]^ folate and 1‐carbon pathway metabolites,^[^ ^41^, ^42^ ^]^ phosphatidylcholines^[^ ^43^ ^]^ and glycerol hydroxybutyrates.^[^ ^28^, ^44^, ^45^ ^]^ Strikingly, there was little evidence for elevation of these metabolites neither in response to hypoxia stimulation alone nor upon LNA‐NC treatment (Figure 5D,E). Thus, these data suggest that LNA‐1a/15b serves to very specifically reprogram the cardiac transcriptome and metabolome to enable and support cardiac tissue regeneration.

### 2.6 Discussion and Conclusion

miRNAs are known to play critical roles in cardiac pathophysiology, including MI and heart failure. Most studies have primarily focused on the role of individual miRNAs, although combinatorial activities of miRNAs are being increasingly investigated and recognized in recent years.^[^ ^46^, ^47^ ^]^ Although the impact of individual miRNA modulation cannot be underestimated, we speculate that more complex processes might necessitate the simultaneous combinatorial modulation of two or more regulatory moieties. Given the complexity and robustness engrained within adult cardiomyocytes to maintain terminal differentiation, structural integrity within the tissue complex, and interaction with surrounding cardiomyocytes, stromal and mural cells in order to facilitate cardiac contractility and function, our observations indicate that the induction and re‐entry of post‐mitotic adult cardiomyocytes could be one such example to benefit from complex and combinatorial miRNA modulation. This is supported by recent data where combinatorial modulation of coding genes through simultaneous co‐expression of four genes (comprising transcription factors and cell‐cycle regulator combinations) have been shown necessary to drive adult cardiomyocyte mitotic re‐entry.^[^ ^48^, ^49^, ^50^ ^]^ Although these findings are of key importance in understanding mechanisms underlying the induction of adult cardiomyocyte mitosis, translating these findings into a clinically feasible strategy is challenging due to the need for cardiac‐directed gene therapy (necessitating the need for invasive delivery procedures) and the clear risk of therapy leakiness which could lead to neoplasm formation in non‐cardiac tissue (due to the ubiquitous nature and function of the transcription factors and cell‐cycle regulator combinations ectopically expressed in these strategies^[^ ^48^, ^49^, ^50^ ^]^). Thus, developing clinically feasible and accessible therapeutic strategies for cardiac regeneration remains an unmet need. Combinatorial miRNAs therapy targeting cardiac enriched miRNAs may offer such an avenue by virtue of the ability to inactivate these miRNAs through systemic delivery of stabilized anti‐sense moieties, thus removing the need for invasive procedures such as catheter‐based delivery or direct cardiac injection.^[^ ^51^, ^52^ ^]^

According to the Human microRNA Disease Database (HMDD) microRNA–disease associations, the 17 miRNAs we selected are upregulated in the patients with MI and heart failure.^[^ ^21^ ^]^ This is consistent with our data demonstrating the elevation of all 17 miRNAs heart biopsies of patients with myocardial infarction (Figure S1A, Supporting Information). Among the proliferation‐regulating miRNAs, we have used a combinatory approach to identify a combination that could further increase cardiomyocyte proliferation compared to individual miRNA. Our study identified that simultaneous inhibition of miRNA‐1a and miRNA‐15b increased cardiomyocyte proliferation and improved cardiac function in vitro and in vivo. Both of miRNA‐1a and miRNA‐15b are highly expressed in hearts, and they have been documented to regulate cardiomyocyte proliferation.^[^ ^53^, ^54^ ^]^ Overexpression of miRNA‐1 in the developing hearts leads to decreased cardiomyocyte proliferation.^[^ ^55^, ^56^ ^]^ Conversely, miRNA‐1a deletion increases cardiomyocyte proliferation in the adult hearts,^[^ ^54^ ^]^ and attenuates cardiac I/R injury in mice and rats.^[^ ^57^, ^58^ ^]^ miRNA‐15b is upregulated in the infarcted zone of porcine cardiac tissue in response to ischemic injury, and miRNA‐15b inhibition increases cardiac regeneration and protects against ischemic‐induced injury.^[^ ^59^ ^]^ Our data showed that combinatory inhibition of miRNA‐1a and miRNA‐15b could further increase cardiomyocyte proliferation in P1 and P7 rat cardiomyocytes, and could enhance cardiomyocyte proliferation and improve cardiac function in mice in vivo. As access to cardiac tissue, in particular, is challenging due to ethical concerns, human cardiac organoids serve as proxy material in which the functionality of this therapeutic approach can be assessed. In utilizing iPSC‐derived human cardiac mimics, we were able to demonstrate a conservation of function of inhibition of miRNA‐1a and miRNA‐15b in driving adult human cardiomyocyte proliferation, leading to maintenance of cardiac contractility in the face of hypoxic insult. Therefore, combinatorial miRNAs therapy may provide therapeutic benefits by simultaneously interfering with the many dysregulated pathways in myocardial infarction and heart failure. In sum, the present finding: i) LNA‐1a/15b synergistically enhanced cardiomyocyte proliferation in vitro and in vivo; and ii) LNA‐1a/15b synergistically reduced MI‐induced EF and fibrosis, and improved cardiac function in mice in vivo; and iii) LNA‐1a/15b synergistically increased cardiomyocyte proliferation and improved contractility in human cardiac mimetics. Our study thus reveals a novel mode of two‐miRNA combination that offers synergistic regulation on cardiac regeneration and enhanced cardiac function in mice following MI.

miRNA‐1 and miRNA‐15b are expressed in a highly tissue‐restricted manner. miRNA‐1 and miRNA‐15b are predominantly expressed in mouse and human heart ventricle, with low levels detected in other tissues.^[^ ^60^, ^61^ ^]^ In the present study, the use of LNA‐modified anti‐miRs in targeting miRNA‐1a and miRNA‐15b, facilitates indirect tissue‐targeting due to the predominant expression of miRNA‐1a and miRNA‐15b in heart tissue. In accord, macroscopic analysis of morphology and structure of non‐cardiac tissue, including the lung, spleen, SKM, liver, and WAT revealed negligible differences between LNA‐1a/15b and LNA‐NC treated mice. Taken together, the synergism offered through simultaneous inhibition miRNA‐1a and miRNA‐15b, combined with the restricted expression of miRNA‐1a and miRNA‐15b, may facilitate tissue‐specific precision therapy, to lower side effects and the possibility of neoplasm formation in other tissues. Thus, inhibiting miRNA‐1a and miRNA‐15b is a promising strategy to enhance cardiac regeneration in patients with heart failure.

Currently, the LNA‐based anti‐miRs have been widely used to suppress the function of miRNAs, and have also been shown to be safe and effective in human studies.^[^ ^11^, ^62^, ^63^ ^]^ Various studies have convincingly shown that targeting miRNAs may improve cell therapy or enhance endogenous cardiac repair processes, and LNA‐based anti‐miRs have already been used in large animal models and a first clinical trial. For example, LNA‐anti‐miR‐21 treatment reduces cardiac fibrosis and hypotrophy and improves cardiac function in a pig model of Ischemia/reperfusion (I/R) injury.^[^ ^64^ ^]^ In addition, LNA‐based anti‐miR‐132 treatment improves EF and ameliorates cardiac dysfunction in response to MI in a porcine model.^[^ ^62^ ^]^ Furthermore, a recent clinical trial involving the delivery of a synthetic LNA‐based antisense oligonucleotide against miRNA‐132 (anti‐miR‐132) in heart failure patients (NCT04045405) revealed a potential benefit of CDR132L in attenuating heart failure.^[^ ^62^ ^]^ MRG‐110, an LNA‐based antisense oligonucleotide targeting miRNA‐92a, has been also tested in healthy human adults and MRG‐110 treatment reduces miRNA‐92a levels and de‐represses the target genes in human peripheral blood cells.^[^ ^11^ ^]^ These studies indicate that further development of LNA‐based anti‐miR therapies could provide a route to developing efficacious therapies, of which LNA‐anti‐miR‐1a/15b would be one such strategy. Moreover, unlike approaches that target individual miRNAs, this dual inhibition addresses multiple regulatory pathways simultaneously, leading to a synergistic enhancement of cardiomyocyte proliferation and improved cardiac function post‐myocardial infarction. This approach not only enhances the cardiac regenerative capacity but also minimizes potential off‐target effects due to the heart‐specific expression patterns of these miRNAs. Furthermore, by using two LNAs, the dose of each LNA is lowered, thus reducing off‐target effects and improving robustness by simultaneously modulating multiple key pathways. This is in line with strategies used in cancer therapy, where combination treatments help overcome resistance, increase efficacy, and provide more comprehensive pathway inhibition. Given the parallels to combination therapies in oncology, this strategy may serve as a model for future regenerative medicine approaches beyond cardiology. Its success in myocardial infarction treatment could pave the way for similar interventions in other tissues where regenerative capacity is limited, such as the central nervous system.

However, several limitations must be addressed before translating this strategy into clinical applications. Most current studies are limited to animal models and human cardiac organoids, and the safety and efficacy of miR‐1 and miR‐15b inhibition in humans remain uncertain. Potential off‐target effects and the long‐term consequences of miRNA inhibition are not fully understood. Future research should focus on conducting comprehensive clinical trials to evaluate the therapeutic potential and safety profile of this approach in humans. Furthermore, variability in patient responses must be considered. Differences in genetic backgrounds, comorbidities, and disease progression could influence the effectiveness of miRNA inhibition, necessitating personalized therapeutic strategies. Biomarker‐based patient selection and stratification may improve treatment outcomes and reduce the risk of adverse effects. Additionally, exploring the underlying molecular mechanisms of miR‐1a and miR‐15b in cardiac biology will be crucial for optimizing therapeutic strategies and minimizing adverse effects.

Taken together, combinatorial LNA‐anti‐miR‐1a/15b treatment results in pro‐proliferative effects on adult cardiomyocytes and improved cardiac function in response to MI. Our data provides a novel and clinically feasible LNA‐based anti‐miR‐1a/15b strategy for the treatment of STEMI, and potentially heart failure through the re‐activation of adult cardiomyocyte mitotic re‐entry and cardiac regeneration.

## 3 Experimental Section

All data produced in the present study are available upon reasonable request to the authors.

### Animals

C57BL/6J mice were obtained from Janvier (Le Genest Saint‐Isle, France). All animals used in this experiment were housed in a temperature‐controlled room with a 12‐h light‐dark cycle and were allowed free access to food and water in agreement with NIH animal care guidelines, §8 German animal protection law, German animal welfare legislation, and with the guidelines of the Society of Laboratory Animals (GV‐SOLAS) and the Federation of Laboratory Animal Science Associations (FELASA). Twelve‐week‐old C57BL/6J mice were subjected to MI surgery, and LNA delivery was performed 4 h after MI. Mice were killed and hearts isolated at the end of the experiment. All protocols were approved by the ethics committee of the regional council (License number FU1201, Regierungspräsidium Darmstadt, Hesse, Germany).

### Human Heart Biopsies

Human heart biopsies were provided by the Institute of Legal Medicine (Goethe University Hospital, Frankfurt am Main, Germany). The human heart biopsies were conducted in compliance with the local ethics committee (license number 116/14 from Goethe University). Samples from healthy hearts as control and from hearts with macroscopically visible signs of acute cardiac infarction as MI hearts and total miRNA were extracted.

### Myocardial Infarction (MI)

MI was performed in 12‐week‐old male C57BL/6J mice and was induced by permanent ligation of the left anterior descending coronary artery (LAD). The mice were monitored up to 28 days after surgery, and the heart dimensions and cardiac function were determined by echocardiography on days 0, 7, 14, 21, and 28. 4 h after MI, in vivo miRCURY LNA Power Inhibitors were injected intraperitoneally (i.p.) into mice at a dose 10 mg kg^−1^. The following in vivo miRCURY LNA Power Inhibitors were purchased from QIAGEN: LNA‐miR‐1a‐3p (339203 YCI0200659‐FZA), LNA‐miR‐15b‐5p (339203 YCI0200641‐FZA), and negative control LNA (339203 YCI0200578‐FZA).

### Isolation and Maintenance of Primary Neonatal Rat Cardiomyocytes

Mated female Sprague Dawley rats were obtained from Janvier (Le Genest Saint‐Isle, France). Primary neonatal rat cardiomyocytes (NRCs) were isolated from post‐natal day 1 (P1) and P7 rat pups by using the neonatal heart dissociation kit (Miltenyi Biotec) and the gentleMACS Dissociator according to the manufacturer's instruction. Isolated cells were pre‐plated in a plating medium (DMEM high glucose, M199 EBS (BioConcept), 10% horse serum, 5% fetal calf serum, 2% L‐glutamine and 1% penicillin/streptomycin) for 75 min in 10 cm cell culture dishes (Nunc) at 37 °C and 5% CO_2_ in a humidified atmosphere to get rid of fibroblasts as described previously.^[^ ^32^ ^]^ NRCs were plated on Type‐I bovine Collagen‐coated (Advanced Biomatrix) plates or dishes in the plating medium. 24 h after isolation of NRCs, the plating medium was changed to a maintenance medium (DMEM high glucose, M199 EBS, 1% horse serum, 2% L‐glutamine, and 1% penicillin/streptomycin).

### Isolation and Maintenance of Primary Rat Fibroblasts

Primary rat fibroblasts were isolated from P1 rat hearts. After pre‐plating the cells in 10 cm cell culture dishes for 75 min, the suspension cells were harvested as cardiomyocytes, the attached cells will be digested and harvested as fibroblasts. The fibroblasts were cultured in DMEM medium with 10% FBS, 2% L‐glutamine, and 1% penicillin/streptomycin at 37 °C and 5% CO_2_ in a humidified atmosphere.

### Preparation and Maintenance of Human iPSC‐CMs

Human induced pluripotent stem cells (hiPSCs) were purchased from Cellular Dynamics International (CMC‐100‐010‐001) and cultured as recommended by the manufacturer. The human iPSC‐derived cardiomyocytes (hiPSC‐CMs) were reprogrammed using the STEMdiff Cardiomyocyte Differentiation Kit (STEMCELL Technologies) according to the manufacture's protocol. Briefly, human iPS cells were plated at a cell density of 3.5 × 10^5^ cells/well on Matrigel‐coated 12‐well‐plates using mTeSR medium supplemented with 5 µM ROCK inhibitor (Y‐27632, STEMCELL Technologies) for 24 h. After 1 day (‐1), the medium was replaced with fresh TeSR medium. To induce cardiac differentiation, the TeSR medium was replaced with Medium A (STEMdiff Cardiomyocyte Differentiation Basal Medium containing Supplement A) at day 0, Medium B (STEMdiff Cardiomyocyte Differentiation Basal Medium containing Supplement B) at day 2, Medium C (STEMdiff Cardiomyocyte Differentiation Basal Medium containing Supplement C) at day 4 and day 6. On day 8, the medium was switched to STEMdiff Cardiomyocyte Maintenance Medium with full medium changes every 2 days, to promote further differentiation into mature cardiomyocyte cells. All experiments were performed in the hiPSC‐CMs at day 40. Hypoxic condition was achieved by using the Hypoxia chamber and the hiPSC‐CMs were cultured at 1% O_2_ for 2 days.

### Human Cardiac Organoids Formation Technique

Human cardiac organoids were created by hiPSC‐CMs. Aggrewell 800 microwell culture plates were used to create the human cardiac organoids in STEMdiff Cardiomyocyte Support Medium (STEMCELL Technologies). Approximately 900 000 wells in 2 mL cell suspension were pipetted into each well. After 2 days of culture, the medium was switched to STEMdiff Cardiomyocyte Maintenance Medium for long‐term culture.

### EdU Labeling In Vitro

The Click‐iT EdU Cell Proliferation Kit for Imaging, Alexa Fluor 488 dye (C10337, Invitrogen, CA, USA), Alexa Fluor 647 dye (C10640, Invitrogen, CA, USA), and Click‐iT Plus EdU Cell Proliferation Kit for Imaging were used in the study according to the manufacturer's protocol. The NRCs were labeled by 2 µM EdU for 2 days, the rat fibroblasts were labeled by 2 µM EdU for 4 h, and the hiPSC‐CMs were labeled by 2 µM EdU for 6 h.

### Contractility Measurement

Every single human cardiac mimetic was transferred into 96‐well‐plate and treated with either LNA‐1/15b or LNA‐NC. Hypoxic condition was achieved by using the Hypoxia chamber and cardiac organoids were cultured at 1% O_2_ for 3 days, then the contractility will be analyzed by IonOptix system. Units/pixels were determined by calibrating the system with a micrometer.

### Calcium Transient Measurements

Human cardiac organoids were cultured in a 96‐well plate and incubated with 2 µM Cal‐520 AM (AAT bioquest) with 0.04% Pluronic F‐127 (AAT bioquest) as previously described.^[^ ^33^ ^]^ After incubation in the incubator at 37 °C for 90 min, the cardiac organoids were rinsed with PBS and medium, then the cardiac organoids were transferred to the 384 well U‐bottom plate and incubated at 37 °C for 1 h. Relative fluorescence units (RFU) were measured using a fluorescence plate reader.

### In Vitro Administration of Locked Nucleic Acids (LNA)

miRCURY LNA Power Inhibitors were directly added to the cell culture medium at a final concentration of 50 nM. All miRCURY LNA Power Inhibitors and negative control LNA were purchased from QIAGEN: LNA‐miR‐1a‐3p (1999990‐801), LNA‐miR‐1‐3P (YI04100840‐AFA), LNA‐miR‐15a‐5p (339146 YCI0200640‐FDA), LNA‐miR‐15b‐5p (339146 YCI0200641‐FDA), LNA‐miR‐16 (339146 YCI0200647‐FDA), LNA‐miR‐27b‐5p (339146 YCI0200648‐FDA), LNA‐miR‐29a‐3p (339146 YCI0200644‐FDA), LNA‐miR‐34a‐5p (339146 (YCI0200649‐FDA), LNA‐miR‐34b‐5p (339146 YCI0200645‐FDA), LNA‐miR‐34c‐5p (339146 YCI0200650‐FDA), LNA‐miR‐92a‐3p (339146 YCI0200646‐FDA), LNA‐miR‐132‐3p (1999990‐801), LNA‐miR‐133a‐3p (1999990‐801), LNA‐miR‐155‐3p (1999990‐801), LNA‐miR‐195a‐5p (339146 YCI0200642‐FDA), LNA‐miR‐208a‐3p (1999990‐801), LNA‐miR‐497a‐5p (339146 YCI0200643‐FDA), LNA‐miR‐873a‐5p (1999990‐801), and negative control LNA CEL‐CONTROL‐INH (339147 YCI0199066‐FFA).

### RNA Isolation, Reverse Transcription, and qRT‐PCR

Samples were harvested in QIAzol Lysis Reagent (QIAGEN), total miRNAs and total mRNAs were isolated with miRNA mini Kit (QIAGEN) according to the manufacturer's protocol. To assess mature miRNA levels, 10 ng total miRNAs were reverse transcript into cDNA using Taqman Advanced miRNA cDNA synthesis kit (Thermo Fisher Scientific) following the manufacturer's instructions. The Applied Biosystems StepOnePlus Real‐Time PCR system (Applied Biosystems, CA, USA) with TaqMan fast Advanced PCR Master Mix (Thermo Fisher Scientific) were used for analysis. TaqManTM Advanced miRNA Assays for mmu‐miR‐1a‐3p (ID: mmu482914_mir), has‐miR‐1‐3p (ID: mmu482914_mir), rno‐miR‐15a‐5p (ID: rno483022_mir), mmu‐miR‐15b‐5p (ID: mmu482957_mir), rno‐miR‐16‐5p (ID: rno481312_mir), rno‐miR‐27b‐5p (ID: rno481016_mir), hsa‐miR‐27b‐sp (ID: 478 789_mir), hsa‐miR‐29a‐3p (ID: 478 587_mir), hsa‐miR‐34a‐5p (ID: rno481304_mir), miR‐34b‐5p (ID: 0 02617), hsa‐miR_34c‐5p (ID: 478 052_mir), miR‐92a‐3p (ID: 000430), rno‐miR‐132‐3p (ID: rno480919_mir), rno‐miR‐133a‐3p (ID: rno481491_mir), mmu‐miR‐155‐3p (ID: mmu481328_mir), mmu‐miR‐195a‐5p (ID: mmu482953_mir), miR‐208a‐3p (ID: 463567‐mat), mmu‐miR‐497a‐5p (ID: mmu482607_mir), miR‐873a‐5p (ID: rno481425_mir), hsa‐miR‐26a‐5p (ID: 477 995_mir), and hsa‐26a‐5p (ID: 000405).

Total RNAs (200 ng) were reverse transcript into cDNA using QuantiTect Reverse Transcription Kit (QIAGEN) according to the manufacturer's protocol. The Applied Biosystems StepOnePlus Real‐Time PCR system (Applied Biosystems, CA, USA) with Fast SYBR Green Master Mix (Thermo Fisher Scientific) were used for analysis. Gene expression levels were normalized against the housekeeping gene *Hprt1*. The following qRT‐PCR primers were used: Mouse: *Nppa*: 5′ GGAGGTCAACCCACCTCTGA 3′ and 5′ CGAAGCAGCTGGATCTTCGT 3′; *Myh6*: 5′ CGGTGACAGTGGTAAAGGCA 3′ and 5′ GGAATGATGCAGCGCACAAA 3′; *Myh7*: 5′ AGAAGAAGGTGCGCATGGAC 3′ and 5′ ATCCTGGCATTGAGTGCATTT 3′; *Tgf‐β2*: 5′TTGTTCCACAGGGGTTAAGG 3′ and 5′ AGCTCGGTCCTTCA GATCCT 3′; *Col3a1*: 5′ CTGTAACATGGAAACTGGGGAAA 3′ and 5′ CCATAGCTGAACTGAAAACCACC 3′; *Thbs1*: 5′ CACCTCTCCGGGTTACTG AG 3′ and 5′ GCAACAGGAACAGGACACCTA 3′; *Hprt*: 5′ CCACTTGTGACGA AAGCACC 3′ and 5′ GTTGTCTACGCTCTGGCAGT 3′.

### Immunofluorescence staining

Immunofluorescence staining was performed as described previously.^[^ ^65^ ^]^ After fixation of cells with 4% paraformaldehyde (PFA)/PBS, the cells were permeabilized and incubated overnight at 4 °C with primary antibodies against sarcomeric α‐actinin (Sigma Aldrich), Aurora B (Abcam), α‐SMA (Abcam), Ki67 (Abcam), pHH3 (Abcam), cleaved (Cl.) Caspase 3 (Abcam) or Tomato lectin (TL) (Sigma Aldrich) diluted in 2% HS/PBS (v/v). After 3 washes with PBS for 5 min, cells were incubated with 4′,6‐diamidino‐2‐phenylindole (DAPI; Thermo Fisher Scientific), AlexaFluor 555 anti‐mouse (Thermo Fisher Scientific) and AlexaFluor 488 anti‐rabbit (Thermo Fisher Scientific) secondary antibody for 1 h at room temperature. Dishes were mounted onto glass slides (Fisher Scientific) with a drop of ProLong Antifade (Thermo Fisher Scientific). Fluorescent images were acquired with the SP5 confocal microscopy (Leica) using a 40x magnification.

Following the α‐SMA, α‐actinin, Wheat–Germ–Agglutinin (WGA), the cells or sections were stained with EdU. EdU staining was conducted using Click‐iT EdU imaging kit (Invitrogen, CA), according to the manufacturer's protocol. Briefly, after incubation with a secondary antibody for 1 h, cells were washed with 3XPBS for 5 min. The cells were then incubated with a Click‐iT reaction cocktail for 30 min for EdU staining, then the cells were washed with 3XPBS for 5min. Finally, fluorescent images were acquired with the SP8 confocal microscopy (Leica) using a 40x magnification.

### ASAT and ALAT Measurement

The blood was collected in tubes prefilled with EDTA for determination of Aspartate aminotransferase (ASAT) and Alanine aminotransferase (ALAT) in sera from animals in different groups. Sera samples were measured in a Cobas 8000 Analyzer (Roche Diagnostics, Germany) using Roche reagents following the manufacturer's instructions.

### Picro Sirius Red Staining

Picro Sirius red staining was used to determine collagen deposition and fibrosis in heart cryosections. 0.1% Picrosirius Red solution was prepared by solving 0.5 g Sirius Red (Waldeck GmbH) in 500 mL picric acid (Sigma‐Aldrich). The cryosections were washed with water and PBS, and then fixed with 4% paraformaldehyde (PFA) for 30min. Then the cryosections were incubated in the 0.1% Picrosirius Red solution for 1 h. After washing two times with acidified water, the sections were dehydrated with 100% ethanol, cleared with xylene, and mounted with Pertex (Medite) as previously described.^[^ ^32^ ^]^

### Metabolomics Sample Preparation

P1 rat cardiomyocytes (5 × 10^5^) were cultured per 3 cm dish. 24 h after isolation miRCURY LNA Power Inhibitors were added to the maintenance medium at a final concentration of 50 nM. The cell culture plates were snap frozen in liquid nitrogen 48 h after miRCURY LNA Power Inhibitors incubation, right after the cells were washed with 75 mM ammonium carbonate (Sigma), adjusted to pH 7.4 with acetic acid. In addition, 20 mg of organ tissues (liver, kidneys, lungs, spleen, and kidneys) were homogenized at full speed. Metabolite extraction from NRC and homogenized tissues was done twice with cold extraction buffer (−20 °C) containing acetonitrile: methanol: water in a 40:40:20 ratio. After centrifugation at full speed, sample extract supernatants were dried by vacuum‐assisted centrifugation.

### Non‐Targeted Metabolomics

Untargeted metabolomics was conducted through metabolite profiling utilizing liquid chromatography high‐resolution tandem mass spectrometry (LC‐HR‐MS/MS). The experimental setup comprised an ultra‐performance liquid chromatography system (Aquity I‐class; Waters GmbH, Eschborn, Germany) coupled with a quadrupole – time‐of‐flight mass spectrometer (QToF) equipped with an additional ion mobility spectrometer (IMS) (VION IMS QToF; Waters GmbH, Eschborn, Germany). Sample extract residues were reconstituted in 200 µL aqueous acetonitrile (10% water) containing 0.1% formic acid and transferred to auto‐sampler glass vials for analysis. A pooled sample, consisting of 20 µL from each individual sample, was prepared separately and analyzed randomly throughout the experiment. Chromatographic separation utilized hydrophilic interaction chromatography (HILIC) with a BEH Amide column (2.1 × 100 mm, 1.7 µm; Waters GmbH, Eschborn, Germany) at 45 °C, employing a gradient of mobile phase A (water with 0.1% formic acid) and mobile phase B (acetonitrile with 0.1% formic acid). The mass range covered mass‐to‐charge ratios between 50 and 1000 Da, with a total scan time set to 0.3 s. Positive and negative electrospray ionization (ESI) in combination with high‐definition data acquisition scan mode (HDMSE) were employed, including ion mobility screening, determination of metabolite‐specific collision cross‐section (CCS) values, accurate mass, and fragment ion mass screenings.

Ion source parameters in positive ESI mode were set to 1 kV and 40 V for capillary voltage and sample cone voltage, and 120 °C and 550 °C for source and desolvation temperature. In negative ESI mode, parameters were adjusted to 1.5 kV and 40 V, and 120 and 450 °C, respectively. Nitrogen was used as cone gas and desolvation gas with flow rates of 50 and 1000 L h^−1^ in positive and negative ESI. Leucine–Enkephalin solution injections were performed for mass correction. Data acquisition and raw data processing were carried out using the Waters Unifi software package 2.1.2. Further data processing involved importing Unifi export files into Progenesis QI software (Nonlinear Dynamics, Newcastle upon Tyne, UK) for peak picking, chromatographic alignment, signal deconvolution, data normalization, and experimental design setup.

For metabolite identification, features obtained from untargeted metabolomics screenings were compared with the Human Metabolome Database (HMDB, www.hmdb.ca) using the Progenesis Metascope plugin, applying a precursor mass accuracy of ≤ 10 ppm and a theoretical fragment mass accuracy of ≤ 10 ppm. Additionally, features were compared to in‐house data obtained by injecting metabolite standards in neat solution, allowing deviations of retention time and CCS of 0.3 min and 3%, respectively. Metabolic features without a metabolite annotation were excluded, and the remaining features, along with their signal intensities and identifications, were exported to Microsoft Excel for further data processing.

### Bioinformatic Analysis

The P1 rat cardiomyocytes were treated with either control LNA‐NC or LNA‐1a/15b in hypoxia with 1% O_2_, and then RNAs were extracted for next‐generation RNA sequencing (RNA‐seq) (Novogene). The integrity and purity of the RNA were assessed, and qualified RNA samples were utilized for PCR amplification in order to construct a cDNA library. The cDNA library was sequenced using an Illumina HiSeq 150 platform. For RNA sequencing analysis, raw RNA‐Seq data (fastq files) were first cleaned using Trimmomatic v0.39 (Trimmomatic: a flexible trimmer for Illumina sequence data). The cleaned reads were then accurately mapped to the Ensembl rat genome (mRatBN7.2) using HISAT 2.2.1, and sequence comparison/mapping format (sam) files were generated.^[^ ^66^ ^]^ The Sam files were then sorted by Samtools v1.19.2 to generate binary comparison/mapping format (bam) files, which HTSeq‐count v2.0.3 uses to summarize gene level counts (HTSeq‐a Python framework for working with high‐throughput sequencing data). DESeq2^[^ ^67^ ^]^ was used to detect differentially expressed genes (DEGs), and the DEGs with the cut‐off criterion (*P* value < 0.05 and Log|FC|>1) were considered for further analysis. The BioSample Database (BioProject ID: PRJNA000000) was selected for the data analysis.

### Statistical Analysis

Data are represented as mean and error bars indicate the standard error of the mean (SEM). Two‐tailed unpaired Student's *t*‐test (Excel) or Mann–Whitney test was used as indicated in the respective figure legends. P values were determined with Prism 9.0 (GraphPad) and *P* < 0.05 was considered statistically significant.

## Conflict of Interest

T.Y., S.D., and J.K. are inventors on a patent application pertaining to the inhibition of miR‐1a and miR‐15b for the treatment of heart disease. The other authors declare that they have no conflict of interest.

## Author Contributions

T.Y. and M.W. equally contributed to this work. T.Y., C.Z., P.M.D., K.B., Y.M., Y.W., A.P.D., and P.M. conducted experiments and analyzed results. H.Y. performed the bioinformatics analyses. S.K. provided the human heart biopsies. Experiments were designed by A.Z., S.D., and J.K. T.Y. and J.K. wrote the manuscript with support from J.W.

## Supporting information



Supporting Information

Supplemental Movie 1

## Data Availability

The data that support the findings of this study are openly available in [Yuan] at [https://doi.org/10.1002/advs.202414455], reference number [6730].
