# Supplementary material for: Targeting miRNA‐1a and miRNA‐15b: A Novel Combinatorial Strategy to Drive Adult Cardiac Regeneration
Source: Adv Sci (Weinh). 2025 Apr 3;12(21):2414455. doi: 10.1002/advs.202414455 (PMC12140343; doi:10.1002/advs.202414455)
Supplement: Supplementary file 1 — Supporting Information [file ADVS-12-2414455-s001.pdf]

ADVANCED SCIENCE
Open Access

Supporting Information

for *Adv. Sci.*, DOI 10.1002/advs.202414455

Targeting miRNA-1a and miRNA-15b: A Novel Combinatorial Strategy to Drive Adult Cardiac Regeneration

*Ting Yuan, Meiqian Wu, Chaonan Zhu, Hao Yu, Minh Duc Pham, Katharina Bottermann, Yijie Mao, Yue Wang, Mathias Langner, Mirko Peitzsch, Arka Provo Das, Silke Kauferstein, Jonathan Ward, Peter Mirtschink, Andreas Michael Zeiher, Stefanie Dimmeler and Jaya Krishnan**

Fig. S1

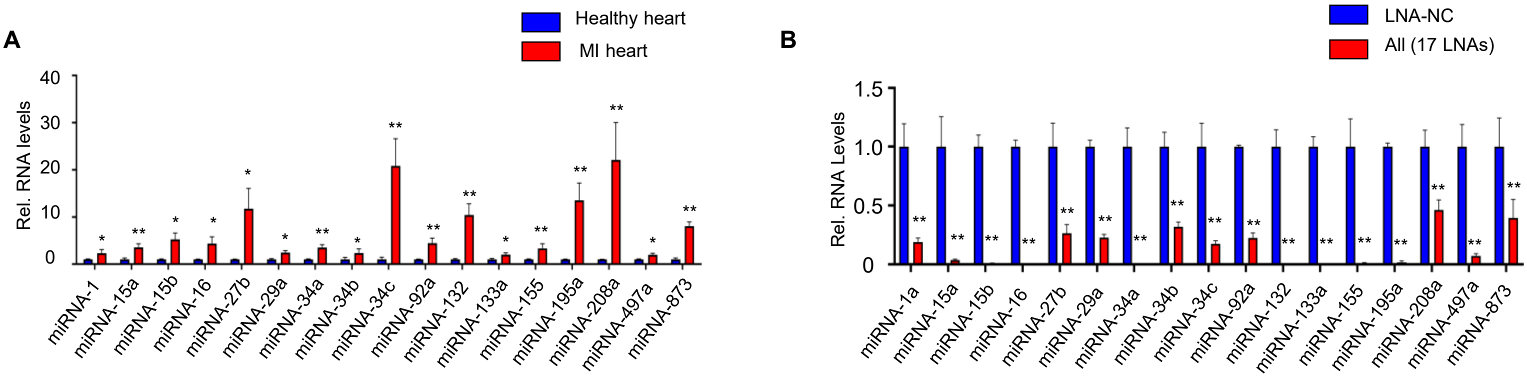

Figure S1. Expression levels of the miRNAs

(**A**) Relative expression of miRNAs in heart biopsies from human patients with MI and healthy controls. Data are expressed as means ± SEM; n=4 for healthy controls, n≥6 for MI group; **P* < 0.05, ***P* < 0.01 vs. healthy heart. All by Mann Whitney test. (**B**) Relative miRNA expression levels were quantified by qRT-PCR in the neonatal P1 rat cardiomyocytes after 48 h treatment with pool of 17 LNAs. Data are expressed as means ± SEM; n=3; **P* < 0.05, ***P* < 0.01 vs. LNA-NC. Two-tailed unpaired t-test.

Fig. S2

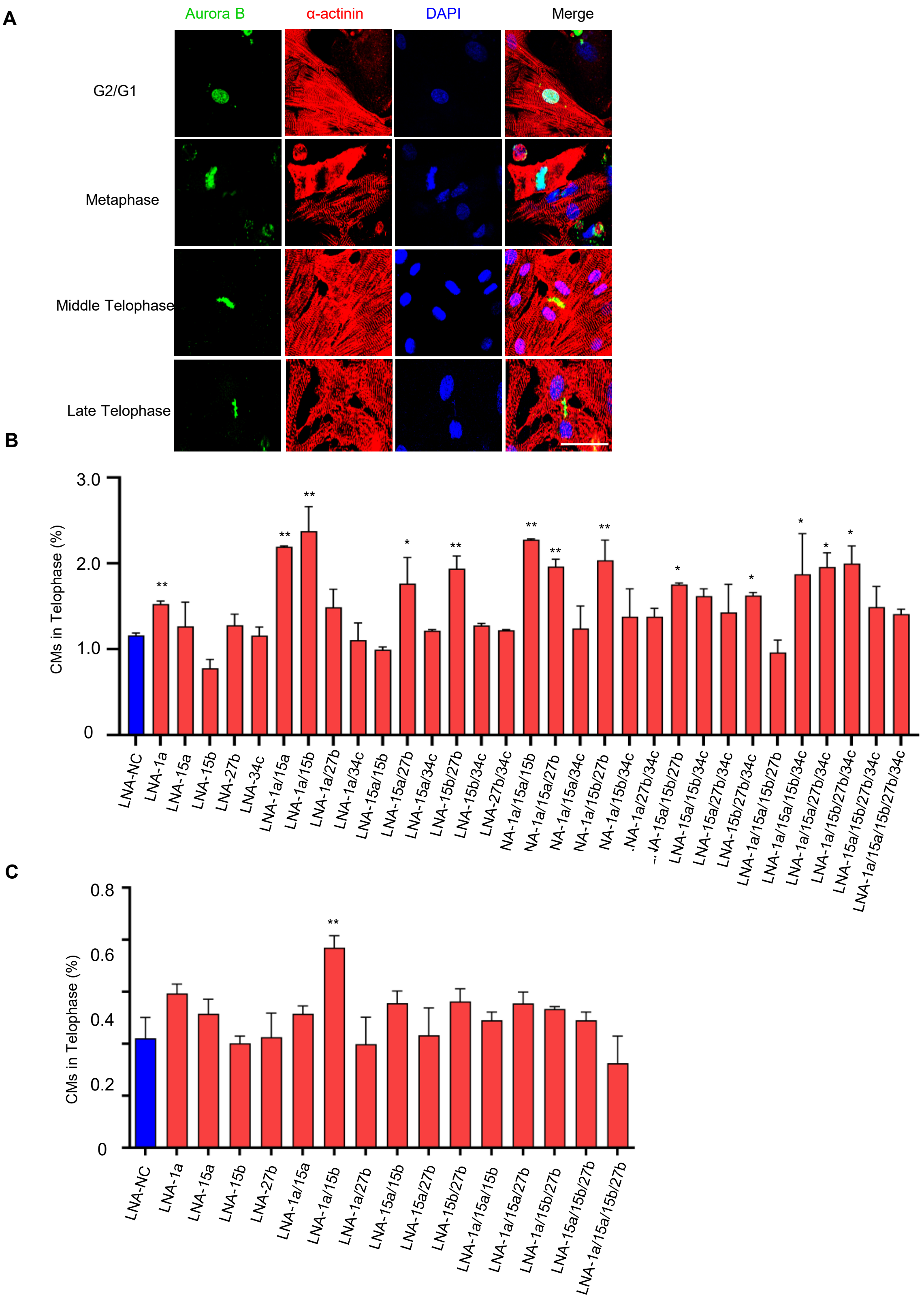

Figure S2. Screening of the co-miRNAi in the rat cardiomyocytes

(**A**) Immunofluorescence images of Aurora B localization at G2/G1 Phase, Metaphase and Telophase. Aurora B (green) and α-actinin (red), and DAPI (blue). Scale bar is 100 µm. (**B**) Bar graph shows the quantification of the percentage of Aurora B^+ cells in telophase in α-actinin$^+$ cardiomyocytes in 31 individual and combinatorial conditions in P1 rat cardiomyocytes. Data are expressed as means ± SEM; n=3; **P* < 0.05, ***P* < 0.01 vs. LNA-NC. Two-tailed unpaired t-test. (**C**) Bar graph shows the quantification of the percentage of Aurora B^+ cells in telophase in α-actinin$^+$ cardiomyocytes in 15 individual and combinatorial conditions in P7 rat cardiomyocytes. Data are expressed as means ± SEM; n=3; **P* < 0.05, ***P* < 0.01 vs. LNA-NC. Two-tailed unpaired t-test.

Fig. S3

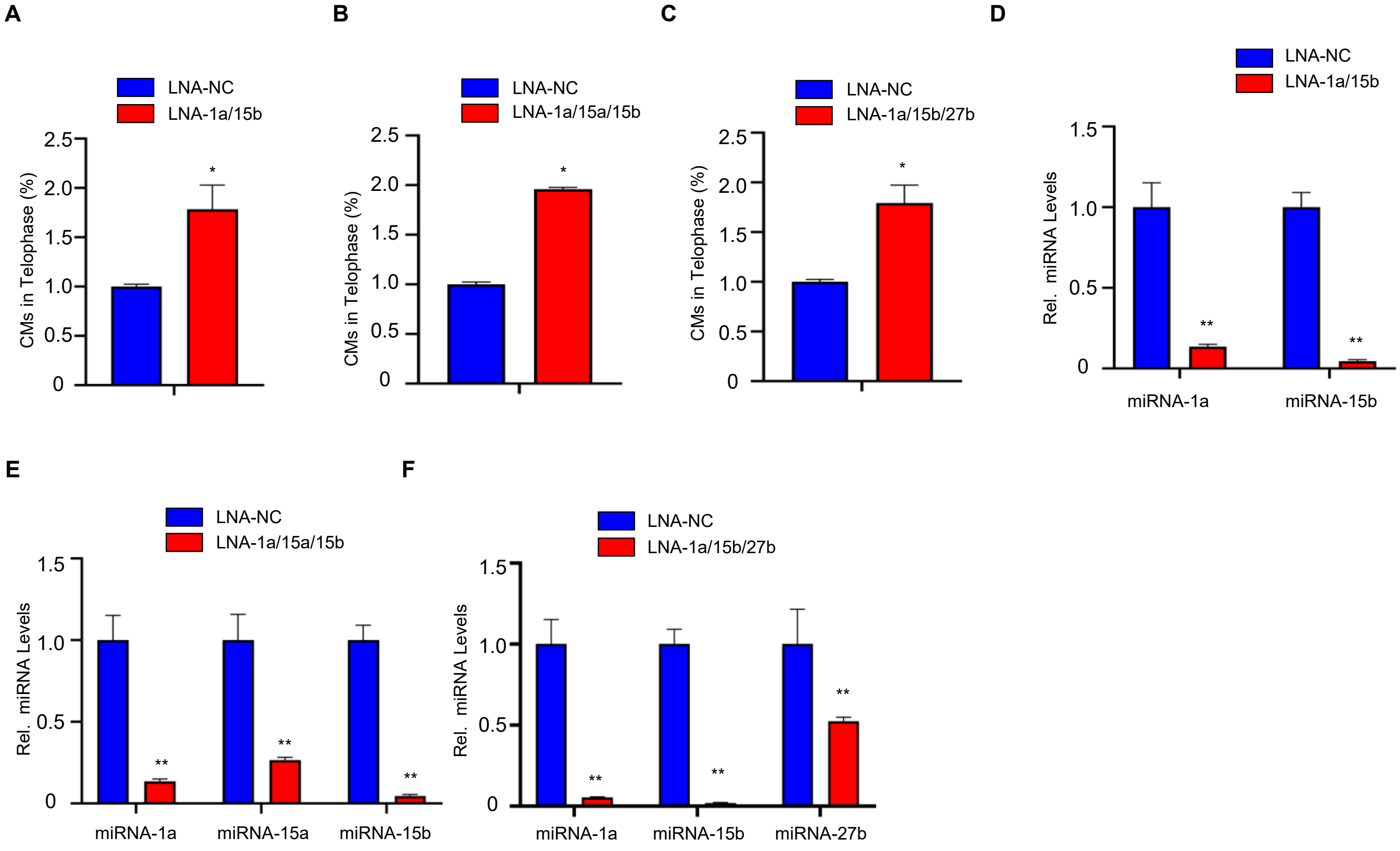

Figure S3. Effect of inhibition of miRNAs in the rat cardiomyocytes

(**A**) Quantification of the percentage of Aurora B^+ cells in α-actinin+ cardiomyocytes for the combination LNA-1a/15b. Data are expressed as means ± SEM; n=3; **P* < 0.05 vs. LNA-NC. Two-tailed unpaired t-test. (**B**) Quantification of the percentage of Aurora B^+ cells in α-actinin+ cardiomyocytes for the combination LNA-1a/15a/15b. Data are expressed as means ± SEM; n=3; **P* < 0.05 vs. LNA-NC. Two-tailed unpaired t-test. (**C**) Quantification of the percentage of Aurora B^+ cells in α-actinin+ cardiomyocytes for the combination LNA-1a/15b/27b. Data are expressed as means ± SEM; n=3; **P* < 0.05 vs. LNA-NC. Two-tailed unpaired t-test. (**D**) Relative miRNA expression levels were quantified by qRT-PCR in the P1 rat cardiomyocytes after 48 h treatment with combination LNA-1a/15b. Data are expressed as means ± SEM; n=3; ***P* < 0.01 vs. LNA-NC. Two-tailed unpaired t-test. (**E**) Relative miRNA expression were levels quantified by qRT-PCR in the P1 rat cardiomyocytes after 48 h treatment with combination LNA-1a/15a/15b. Data are expressed as means ± SEM; n=3; ***P* < 0.01 vs. LNA-NC. Two-tailed unpaired t-test. (**F**) Relative miRNA expression levels were quantified by qRT-PCR in the P1 rat cardiomyocytes after 48 h treatment with combination LNA-1a/15b/27b. Data are expressed as means ± SEM; n=3; ***P* < 0.01 vs. LNA-NC. Two-tailed unpaired t-test.

Fig. S4

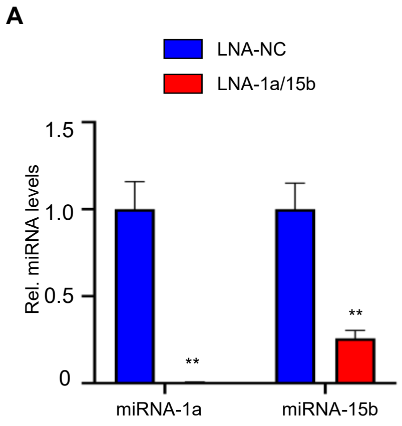

Figure S4. miRNA expression levels in the P7 rat cardiomyocytes

(**A**) Relative miRNA expression levels were quantified by real-time PCR in the P7 rat cardiomyocytes after 48 h treatment with LNA-1a/15b. Data are expressed as means ± SEM; n=3; $^{**}P < 0.01$ vs. LNA-NC. Two-tailed unpaired t-test.

Fig. S5

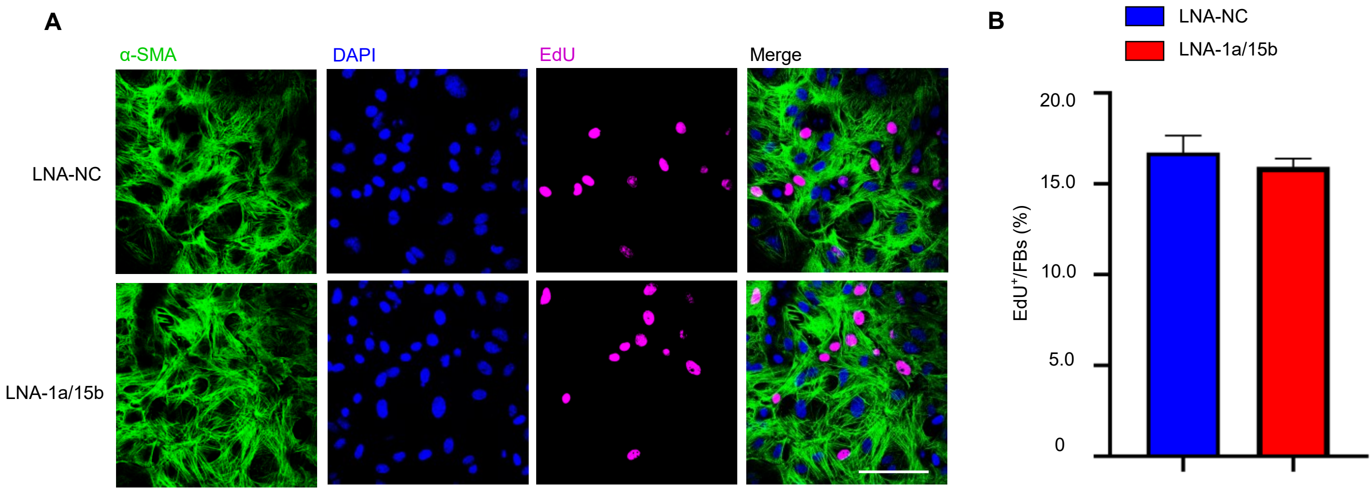

Figure S5. The effects of combination LNA-1a/15b in rat fibroblasts

(**A**) Representative immunofluorescence images of EdU incorporation on rat fibroblasts treated with LNA-1a/15b or LNA-NC. EdU labels proliferating cells (Magenta); α-SMA marks fibroblast (green) and DAPI (Blue). Scale bar is 100 μm. (**B**) Quantification of percentages of EdU^+ cardiomyocytes. Data are expressed as means ± SEM; n=3 per group. Two-tailed unpaired t-test.

Fig. S6

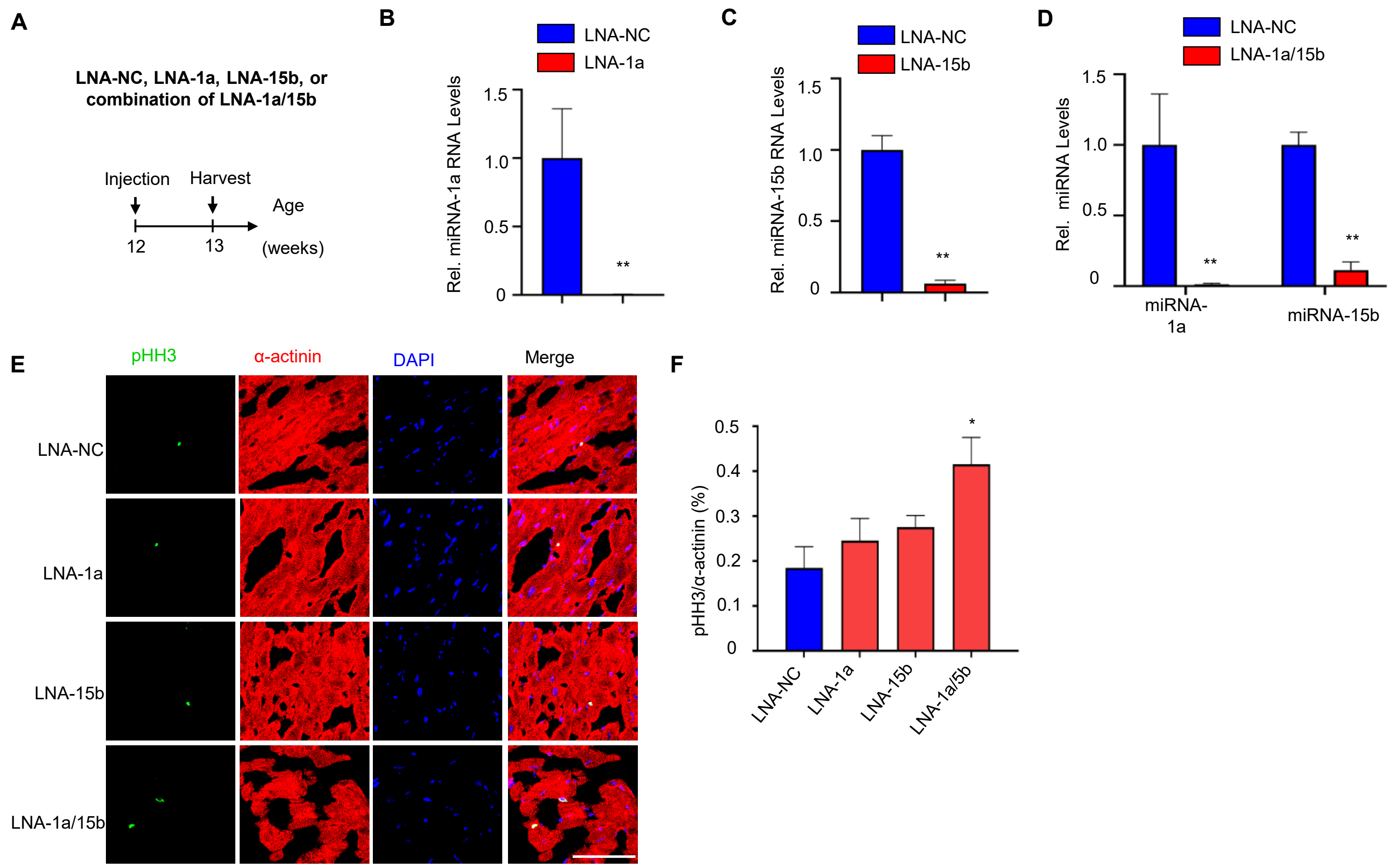

Figure S6. Inhibition of miRNA-1a and/or miRNA-15b by LNA-modified oligonucleotides in mice *in vivo*

(**A**) Schematic representation of the experimental timeline of mice injected with control LNA-NC, LNA-1a, LNA-15b or LNA-1a/15b. The mouse hearts were harvested at day 7 post LNAs injection. (**B-D**) Relative miRNA expression levels were quantified by real-time PCR in the heart after 7 days injection with LNA-NC, LNA-1a, LNA-15b or LNA-1a/15b. Data are expressed as means ± SEM; n=3 mice per group; $^{**}P < 0.01$ vs. LNA-NC. Two-tailed unpaired t-test. (**E**) Representative immunofluorescence images of pHH3 on heart sections of adult hearts injected with LNA-1a, LNA-15b, LNA-1a/15b or control LNA-NC for 7 days. pHH3 labels proliferating cells (green); cardiomyocyte-specific α-actinin (red) and DAPI (Blue). (**F**) Quantification of percentages of pHH3+ cardiomyocytes. Data are represented as Mean ± SEM; n=3 mice per group; $^{*}P < 0.05$. vs. LNA-NC. Two-tailed unpaired t-test.

Fig. S7

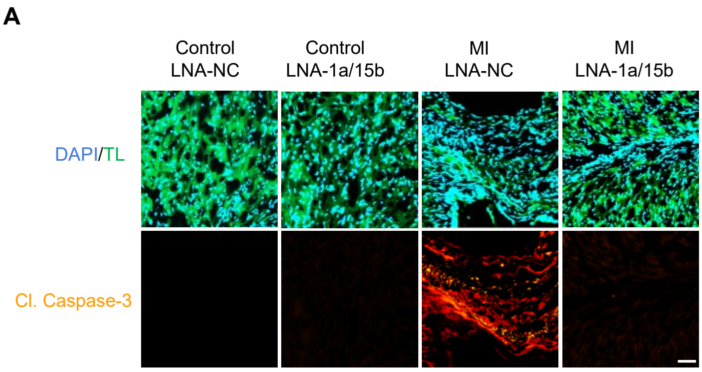

Figure S7. Detection of apoptotic cells by Cl. Caspase 3 and TUNEL staining

(**A**) Immunofluorescence images of Cl. Caspase 3 on heart sections from adult hearts injected with LNA-1a/15b or control LNA-NC 28-days post MI injury. DAPI labels nuclear (Blue); Tomato-lectin (Green) labels compact myocardium; Cl. Caspase 3 (Orange) label apoptotic cells. Scale bar is 100 µm. MI, Myocardial Infarction.

Fig. S8

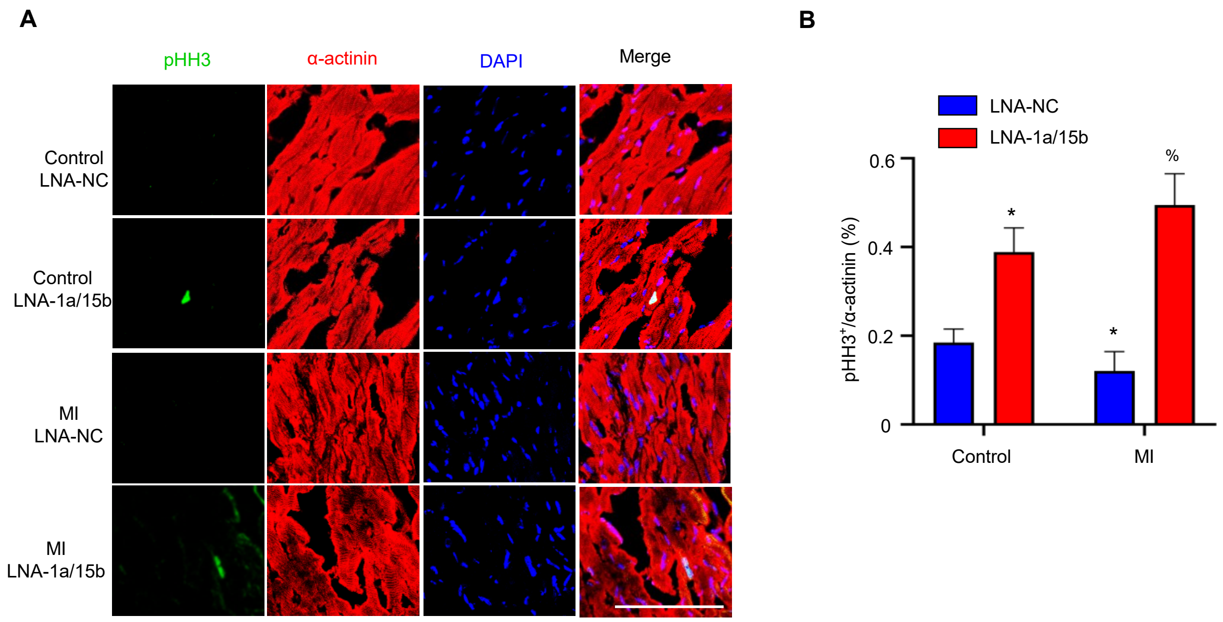

Figure S8. Inhibition of miRNA-1a and miRNA-15b by LNA-1a/15b in mice subjected to Myocardial Infarction (**A**) Representative immunofluorescence images of pHH3 on heart sections of adult hearts injected with LNA-1a/15b or control LNA-NC 28-days post MI injury. pHH3 labels proliferating cells (green); cardiomyocyte-specific α-actinin (red) and DAPI (Blue). Scale bar is 100 μm. (**B**) Quantification of percentages of $pHH3^+$ cardiomyocytes. Data are expressed as means ± SEM; n=3 mice per Control group, n=5 mice per MI group; **P* < 0.05 vs. Control LNA-NC, % *P* < 0.05 vs. MI LNA-NC. Two-tailed unpaired t-test. MI, Myocardial Infarction.

Fig. S9

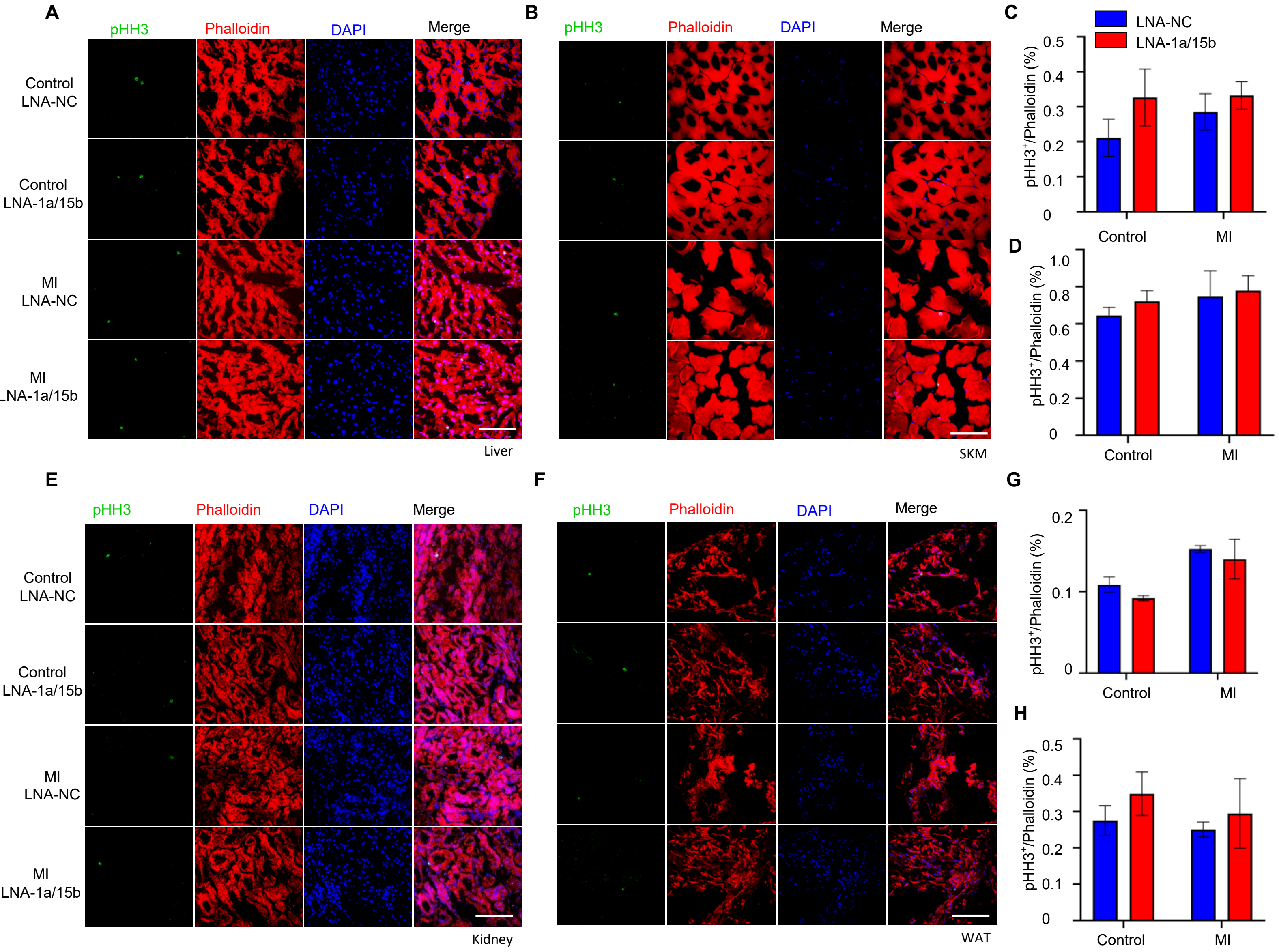

Figure S9. The effects of combinatorial miRNA-1a/15b interference on proliferation on non-cardiac tissues

The liver, SKM, Kidney and WAT tissues from the LNA-NC or LNA-1a/15b treated mice were stained for pHH3 (green), Phalloidin (red) and DAPI (blue). (**A**) Representative images of pHH3 (green), Phalloidin (red) and DAPI (blue) in liver. Scale bar is 100 μm. (**B**) Representative images of pHH3 (green), Phalloidin (red) and DAPI (blue) in SKM. Scale bar is 100 μm. (**C**) Quantification of percentages of $pHH3^+$ liver cells. Data are expressed as means ± SEM; n=3 mice per group. Two-tailed unpaired t-test. (**D**) Quantification of percentages of $pHH3^+$ SKM cells. Data are expressed as means ± SEM; n=3 mice per group. Two-tailed unpaired t-test. (**E**) Representative images of Ki67 (green), Phalloidin (red) and DAPI (blue) in kidney. Scale bar is 100 μm. (**F**) Representative images of pHH3 (green), Phalloidin (red) and DAPI (blue) in WAT. Scale bar is 100 μm. (**G**) Quantification of percentages of $pHH3^+$ kidney cells. Data are expressed as means ± SEM; n=3 mice per group. Two-tailed unpaired t-test. (**H**) Quantification of percentages of $pHH3^+$ WAT cells. Data are expressed as means ± SEM; n=3 mice per group. Two-tailed unpaired t-test. SKM, Skeletal Muscle; WAT, White Adipose Tissue.

Fig. S10

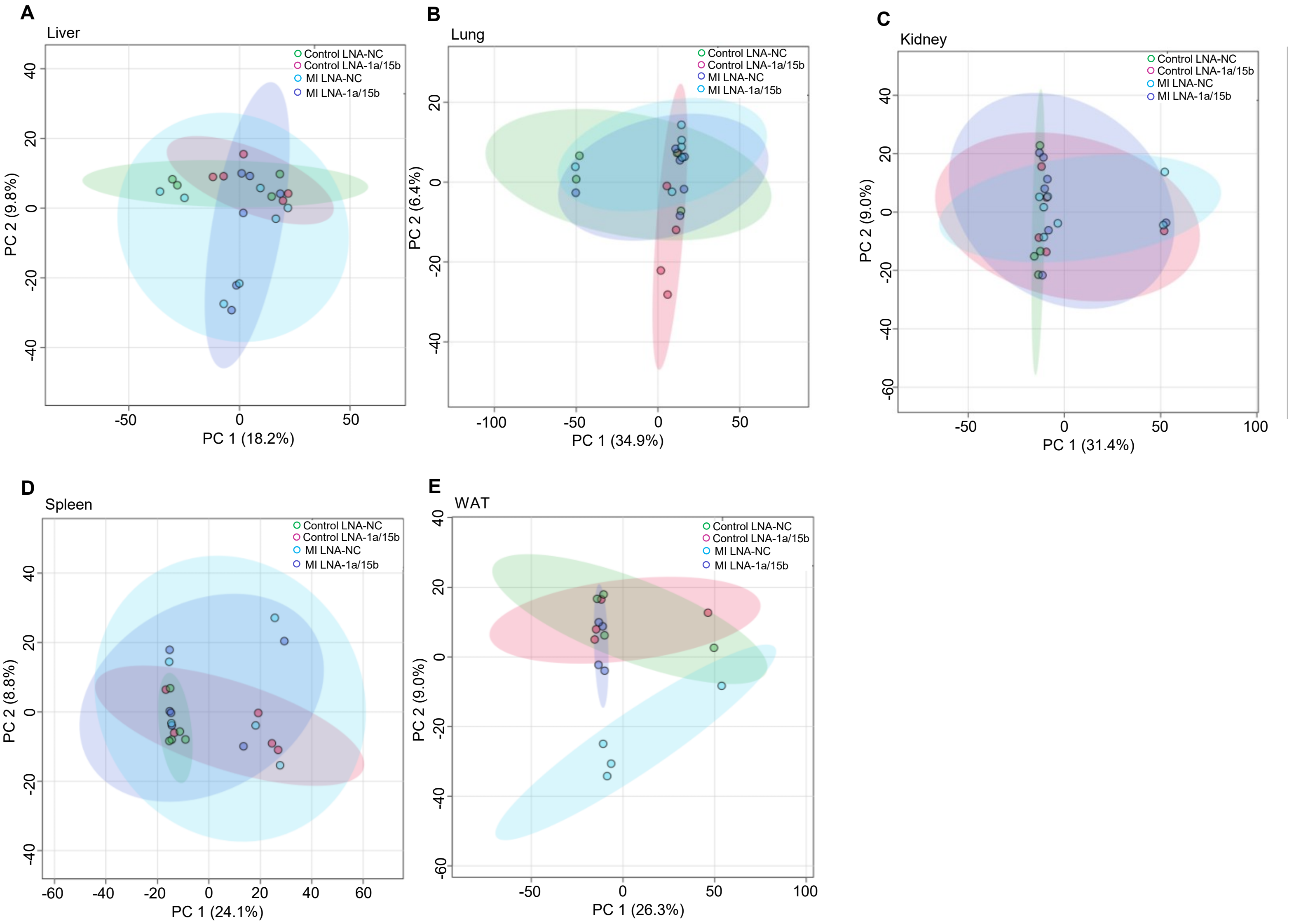

Figure S10. The effects of combinatorial miRNA-1a/15b interference on non-cardiac tissue metabolism

Principal Component Analysis (PCA) of denoted cohorts on Liver (**A**), Lung (**B**), Kidney (**C**), spleen (**D**) and WAT (**E**). Control LNA-NC: n=4; Control LNA-1a/15b: n=4; MI LNA-NC: n=4-7; MI LNA-1a/15b: n=4-6. WAT, White Adipose tissue.

Fig. S11

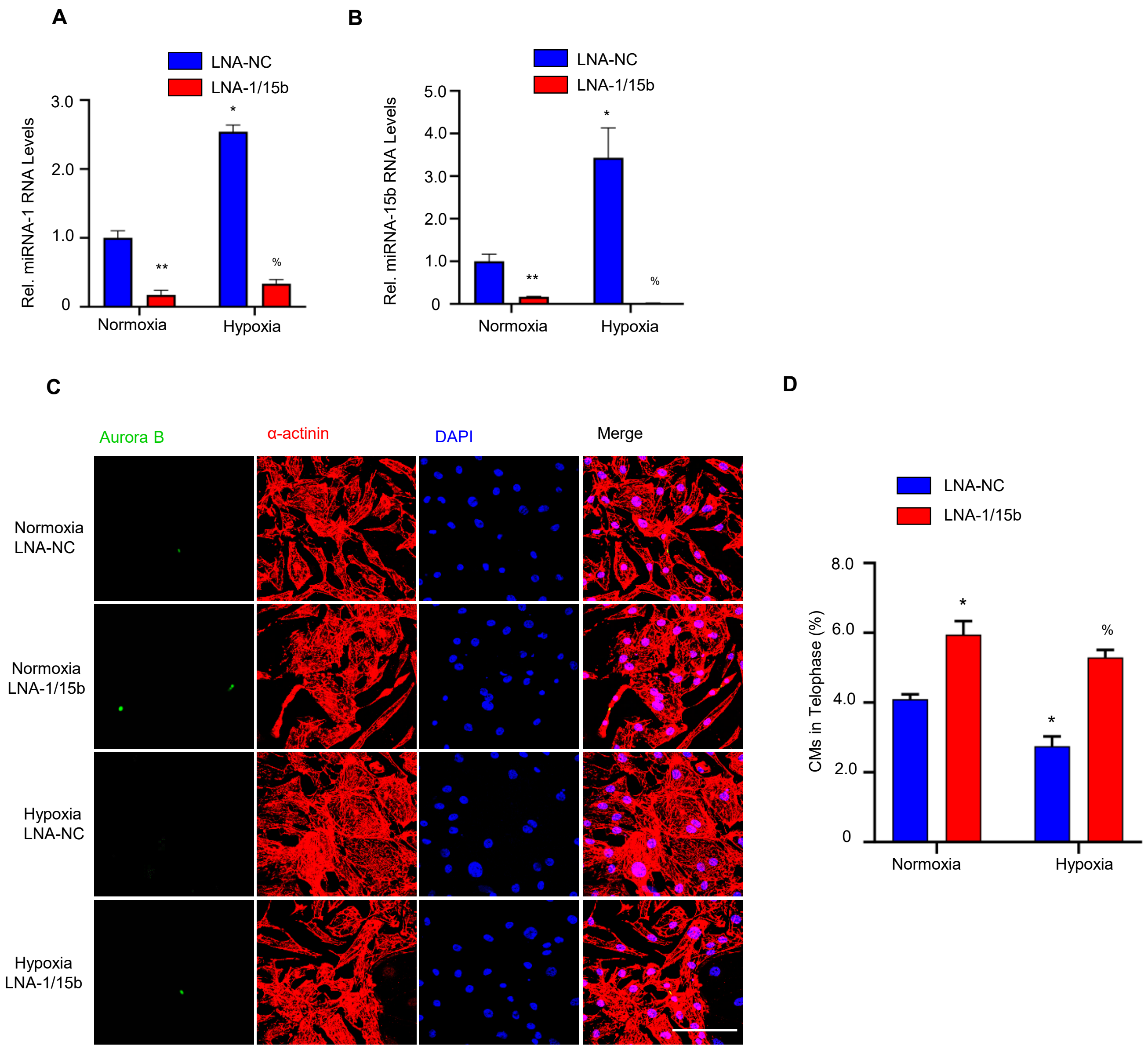

Figure S11. Inhibition of miRNA-1 and miRNA-15b by LNA-1/15b in human iPSC-CMs

(**A**) Relative miRNA-1 expression level was quantified by real-time PCR in the 40-day-old human iPSC-CMs 48h after LNA-1/15b or LNA-NC treatment in both normoxia and hypoxia *in vitro*. Data are expressed as means ± SEM; n=3; **P* < 0.05, ** *P* < 0.01 vs. Normoxia LNA-NC, %*P* < 0.05 vs. hypoxia LNA-NC. Two-tailed unpaired t-test. (**B**) Relative miRNA-15b expression level was quantified by real-time PCR in the 40-day-old human iPSC-CMs 48h after LNA-1/15b or LNA-NC treatment in both normoxia and hypoxia *in vitro*. Data are expressed as means ± SEM; n=3; **P* < 0.05, ***P* < 0.01 vs. Normoxia LNA-NC, %*P* < 0.05 vs. hypoxia LNA-NC. Two-tailed unpaired t-test. (**C**) Representative immunofluorescence images of Aurora B (green), α-actinin (red) and DAPI in 40-day-old human iPSC-CMs 48h after LNA-1/15b or LNA-NC treatment in both normoxia and hypoxia *in vitro*. (**D**) Quantification of percentages of Aurora B^+ in telophase in cardiomyocytes. Data are expressed as means ± SEM; n=3 per group; **P* < 0.05 vs. Normoxia LNA-NC, %*P* < 0.05 vs. Hypoxia LNA-NC. Two-tailed unpaired t-test. Scale bar is 100 μm. Human iPSC-CMs, human-induced pluripotent stem cell-derived cardiomyocytes.

Fig. S12

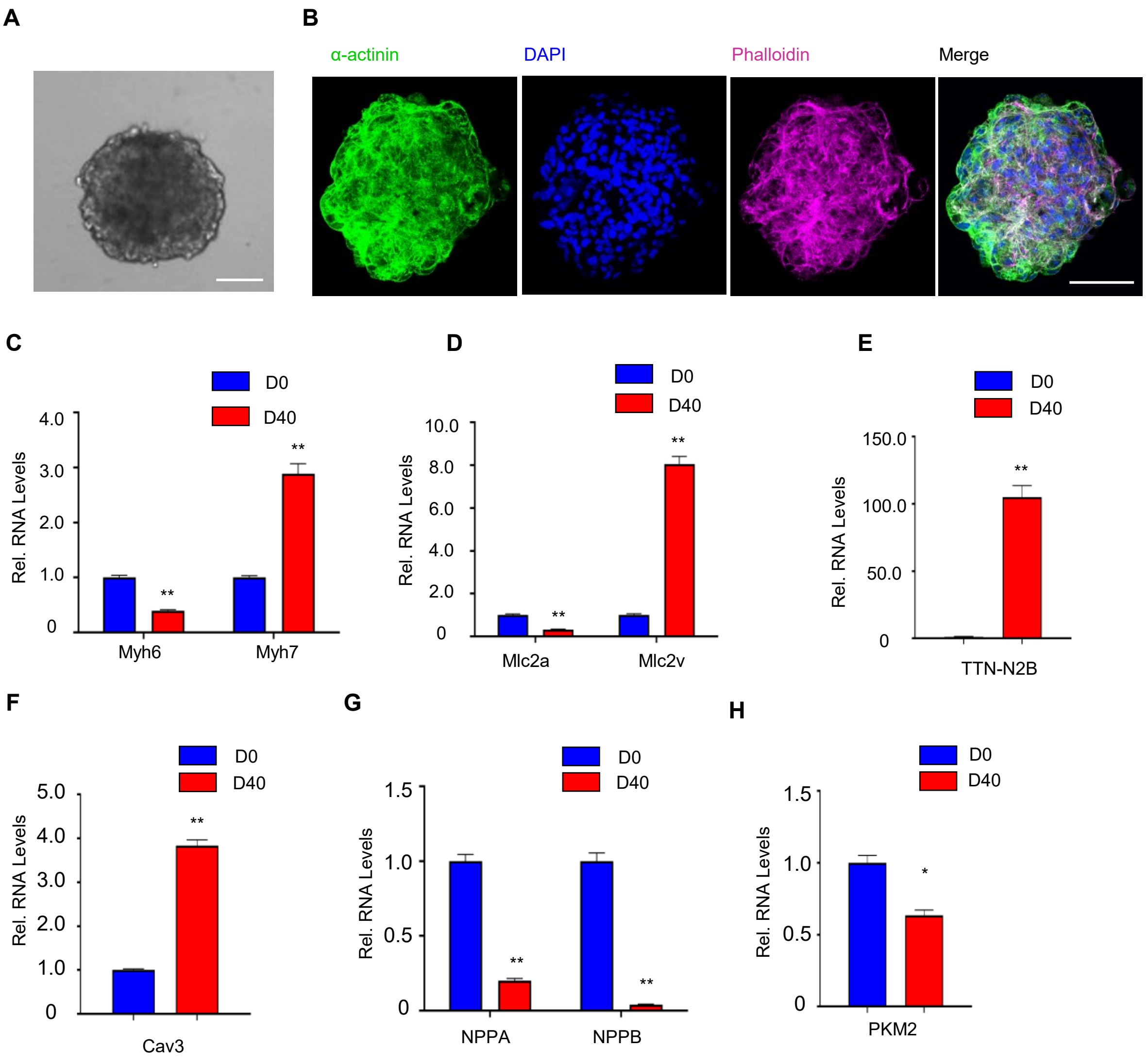

Figure S12. Maturation of human cardiac mimetics

(**A**) Representative brightfield images of 40-day-old human cardiac mimeticss. Scale bar is 100 μm. (**B**) Representative immunofluorescence images of cardiomyocyte-specific α-actinin (green), DAPI (blue) and Phalloidin (Magenta) on whole human cardiac mimetics. Scale bar is 100 μm. (**C-H**) The human cardiac mimetics were harvested at day 0 and day 40, and the gene expression levels including Myh6, Myh7, Mlc2a, Mlc2v, TTN-N2B, Cav3, NPPA, NPPB and PKM2for cardiomyocyte maturation were detected by the qRT-PCR. Data are expressed as means ± SEM; 50 organoids per group; *$P < 0.05$, **$P < 0.01$ vs. D0. Two-tailed unpaired t-test. D, day.

Fig. S13

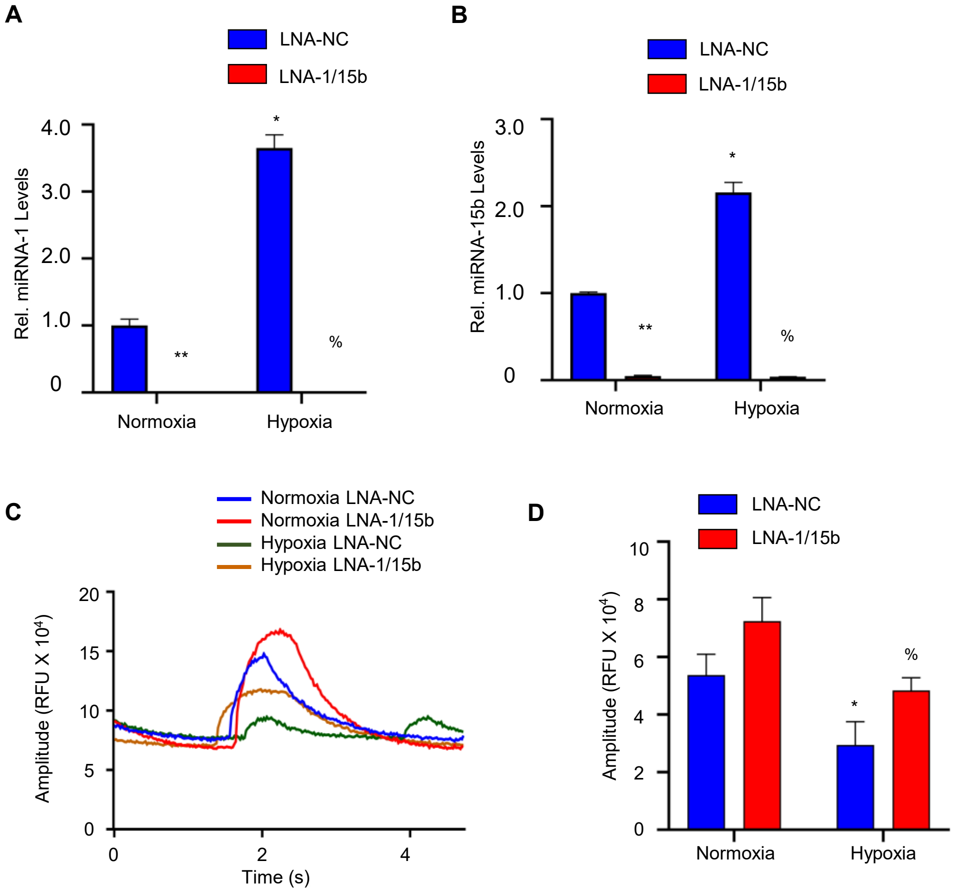

Figure S13. Inhibition of miRNA-1 and miRNA-15b by LNA-1/15b in human cardiac mimetics

(**A**) Relative miRNA-1 expression level was quantified by real-time PCR in the 40-day-old human human cardiac mimetics 96h after LNA-1/15b or LNA-NC treatment in both normoxia and hypoxia *in vitro*. Data are expressed as means ± SEM; n=3; **P* < 0.05, ***P* < 0.01 vs. Normoxia LNA-NC, % *P* < 0.05 vs. Hypoxia LNA-NC. Two-tailed unpaired t-test. (**B**) Relative miRNA-15b expression level was quantified by real-time PCR in the 40-day-old human human cardiac mimetics 96h after LNA-1a/15b or LNA-NC treatment in both normoxia and hypoxia *in vitro*. (**C**, **D**) Human cardiac organoids were treated with LNA-1/15b or LNA-NC treatment in both normoxia and hypoxia in vitro, and then the contractility assay was performed by the calcium transient. (**C**) Raw traces illustrating Cal-520 calcium transients in cardiac organoids. (**D**) Quantification of the RFU. Data are expressed as means ± SEM; 7 - 12 organoids per group; **P* < 0.05 vs. Normoxia LNA-NC, % *P* < 0.05 vs. Hypoxia LNA-NC. Two-tailed unpaired t-test. RFU, relative fluorescence units.

Fig. S14

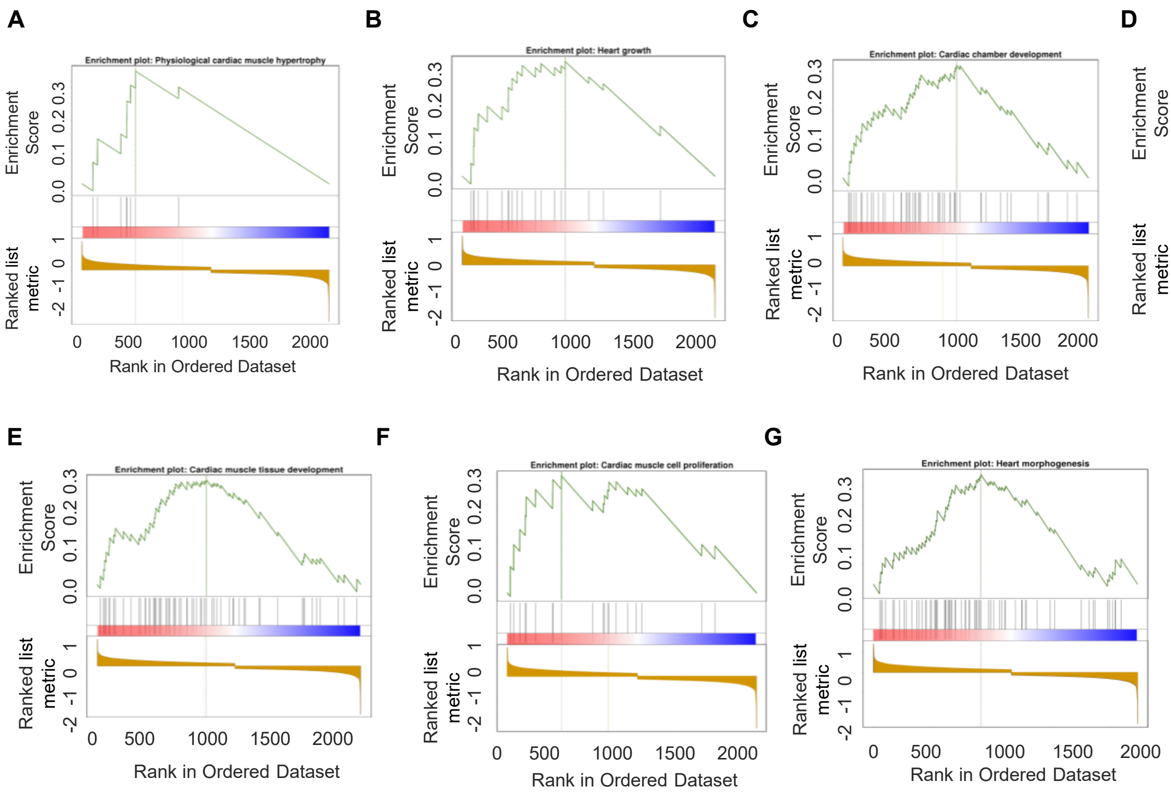

Figure S14. Gene Set Enrichment Analysis (GSEA) enrichment plots related to the heart development

(**A**) GSEA plot for GO class Physiological cardiac muscle hypertrophy in LNA-1a/15b group. (**B**) GSEA plot for Heart growth in LNA-1a/15b group. (**C**) GSEA plot for Cardiac chamber development in LNA-1a/15b group. (**D**) GSEA plot for Cardiocyte differentiation in LNA-1a/15b group. (**E**) GSEA plot for Cardiac muscle tissue development in LNA-1a/15b group. (**F**) GSEA plot for Cardiac muscle cell proliferation in LNA-1a/15b group. (**G**) GSEA plot for Heart morphogenesis in LNA-1a/15b group.

Fig. S15

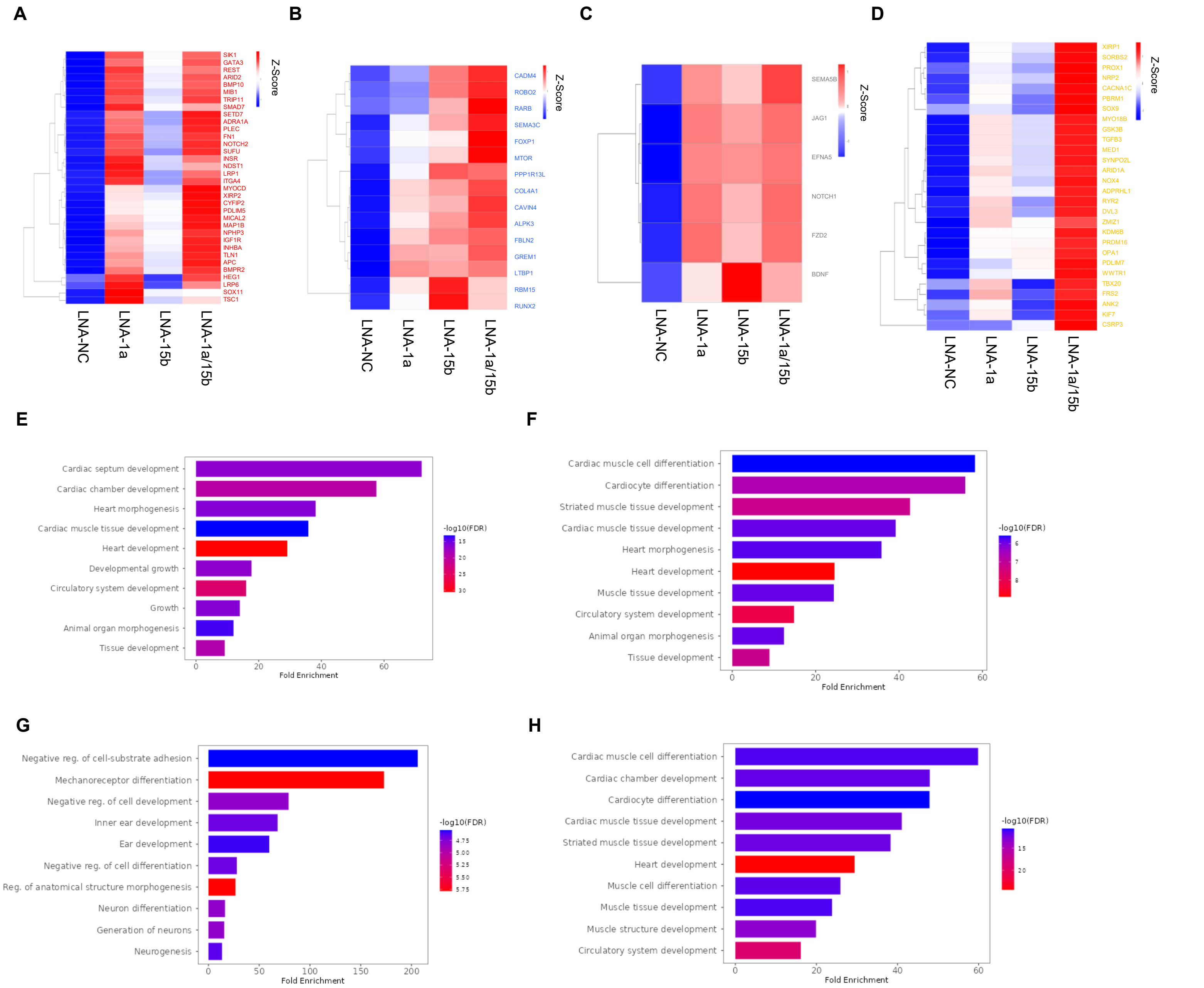

Figure S15. Changes in the gene expression related to heart development with inhibition of miRNA-1a, miRNA-15b and miRNA-1a/15b

(**A-D**) Heat map of RNA-seq expression z-scores computed for heart development related genes that are differentially expressed. Red and blue colors represent higher and lower relative expression levels, respectively. (**A**) Heat map shows that the genes are upregulated specifically by miRNA-1a depletion, but the genes are not significantly upregulated by miRNA-15b depletion. (**B**) Heat map shows that the genes are upregulated specifically by miRNA-15b depletion, but the genes are not significantly upregulated by miRNA-1a depletion. (**C**) Heat map shows that the genes are regulated in common by both miRNA-1a and miRNA-15b depletion. (**D**) Heat map shows that the genes are regulated unique to combinatorial inhibition of miRNA-1a and miRNA-15b, but the genes are unaffected or only mildly altered (and below the threshold of significance) upon miRNA-1a depletion or miRNA-15b depletion alone. Means of n = 3 biological replicates per group. (**E-H**) Enrichment analysis revealed the Gene Ontology (GO) biological process. (**E**) Top 10 GO-biological pathways of upregulated DEGs specifically in miRNA-1a depleted P7 rat cardiomyocytes. (**F**) Top 10 GO-biological pathways of upregulated DEGs specifically in miRNA-15a depleted P7 rat cardiomyocytes. (**G**) Top 10 GO-biological pathways of upregulated DEGs common to both miRNA-1a and miRNA-15b depleted P7 rat cardiomyocytes. (**H**) Top 10 GO-biological pathways of upregulated DEGs unique to combinatorial inhibition of miRNA-1a and miRNA-15b in P7 rat cardiomyocytes. DEGs, Differentially expressed genes.

Video Legends

Video S1. Spontaneous contraction of human cardiac organoids
